# What drives the perceptual change resulting from speech motor adaptation? Evaluation of hypotheses in a Bayesian modeling framework

**DOI:** 10.1371/journal.pcbi.1005942

**Published:** 2018-01-22

**Authors:** Jean-François Patri, Pascal Perrier, Jean-Luc Schwartz, Julien Diard

**Affiliations:** 1 Univ. Grenoble Alpes, CNRS, GIPSA-Lab UMR 5216, F-38000 Grenoble, France; 2 Univ. Grenoble Alpes, CNRS, LPNC UMR 5105, F-38000 Grenoble, France; University of California at Berkeley, UNITED STATES

## Abstract

Shifts in perceptual boundaries resulting from speech motor learning induced by perturbations of the auditory feedback were taken as evidence for the involvement of motor functions in auditory speech perception. Beyond this general statement, the precise mechanisms underlying this involvement are not yet fully understood. In this paper we propose a quantitative evaluation of some hypotheses concerning the motor and auditory updates that could result from motor learning, in the context of various assumptions about the roles of the auditory and somatosensory pathways in speech perception. This analysis was made possible thanks to the use of a Bayesian model that implements these hypotheses by expressing the relationships between speech production and speech perception in a joint probability distribution. The evaluation focuses on how the hypotheses can (1) predict the location of perceptual boundary shifts once the perturbation has been removed, (2) account for the magnitude of the compensation in presence of the perturbation, and (3) describe the correlation between these two behavioral characteristics. Experimental findings about changes in speech perception following adaptation to auditory feedback perturbations serve as reference. Simulations suggest that they are compatible with a framework in which motor adaptation updates both the auditory-motor internal model and the auditory characterization of the perturbed phoneme, and where perception involves both auditory and somatosensory pathways.

## Author summary

Experimental evidence suggest that motor learning influences categories in speech perception. These observations are consistent with studies of arm motor control showing that motor learning alters the perception of the arm location in the space, and that these perceptual changes are associated with increased connectivity between regions of the motor cortex. Still, the interpretation of experimental findings is severely handicapped by a lack of precise hypotheses about underlying mechanisms. We reanalyze the results of the most advanced experimental studies of this kind in speech, in light of a systematic and computational evaluation of hypotheses concerning motor and auditory updates that could result from motor learning. To do so, we mathematically translate these hypotheses into a unified Bayesian model that integrates for the first time speech production and speech perception in a coherent architecture. We show that experimental findings are best accounted for when motor learning is assumed to generate updates of the auditory-motor internal model and the auditory characterization of phonemes, and when perception is assumed to involve both auditory and somatosensory pathways. This strongly reinforces the view that auditory and motor knowledge intervene in speech perception, and suggests likely mechanisms for motor learning in speech production.

## Introduction

The fact that perception has an influence on motor learning is known and has been the focus of a large number of studies. The converse, i.e. that motor learning would influence perception, seems more intriguing and unclear. For speech, shifts in perceptual boundaries have been shown to result from motor learning induced by perturbations of the auditory feedback [1, 2] or perturbations of the articulatory gestures [3]. In the context of the well-known historical debates about the primitives (auditory/articulatory/motor) of speech perception [4–8], these findings could be interpreted as evidence in support of theories assuming the involvement of speech production processes in speech perception. However, an influence of speech motor learning on perceptual categorization of speech sounds does not necessarily imply an involvement of brain motor areas in speech perception. Indeed, the unusual auditory signals experienced during the adaptation process may by themselves be responsible for the observed perceptual shift.

From this observation, and building up on Shiller et al.’s experiment [2], Lametti et al. [1] specifically attempted to disentangle the respective influence of motor functions and altered sensory inputs on the perceptual boundary shifts. To do so, they developed an experimental protocol designed to assess separately the learning effects induced by changes in auditory feedback, on the one hand, and those arising from changes in motor control, on the other hand. They concluded that the origin of the perceptual change is indeed motor rather than sensory.

Lametti et al.’s study is very rich and relies on a solid experimental methodology. However we argue that their reasoning, because it is only qualitative, is incomplete, and does not enable to fully understand the nature of the mechanisms underlying the link observed after motor learning between changes in motor functions and perceptual changes.

In the present work we propose to dig into these questions using a previously defined Bayesian model [9]. This model was previously used to study the relative roles of auditory and proprioceptive representations in speech gesture planning; here we adapt this model to identify, implement and compare different hypotheses concerning motor adaptation. We analyze the consequences of these different hypotheses on perception and production mechanisms and suggest additional tentative interpretations of the experimental findings reported by Lametti et al. [1]. This constitutes, in our view, an important step to better relate experimental data to theories of speech production and speech perception, and further enlighten the possible role of motor processes in speech perception. Importantly, the Bayesian model we use enables to translate classical and transversal questions about motor control, perception, learning and adaptation into computations and predictions. Such a model is a methodological tool to tackle these issues widely in speech production and speech perception, as well as in arm motor control [10, 11].

The body of this paper is divided into four sections. The remaining of this section gives an overview of the main experimental paradigms and facts reported by Lametti et al. [1]. We then present our modeling framework to deal with these experimental findings; this is presented in Section “Model”. The interpretation of the results of simulations are presented in Section “Results”, and discussed in Section “Discussion”.

### Influence of motor learning upon speech perception: Overview of experimental facts

The influence of speech motor learning on speech perception was first reported by Shiller et al. [2] (this study is called “S-09” henceforth). Motor learning was implemented by perturbing the auditory feedback of subjects when they were producing the fricative /s/: it consisted in shifting down the first spectral moment of /s/ in such a way that it sounded more like /∫/. They observed that subjects adapted their articulation after training in order to compensate, partially, for the perturbation, and the perceptual test after adaptation revealed a shift of the perceptual boundary between /s/ and /∫/ toward /∫/ (more sounds were perceived like /s/).

**Fig 1:**
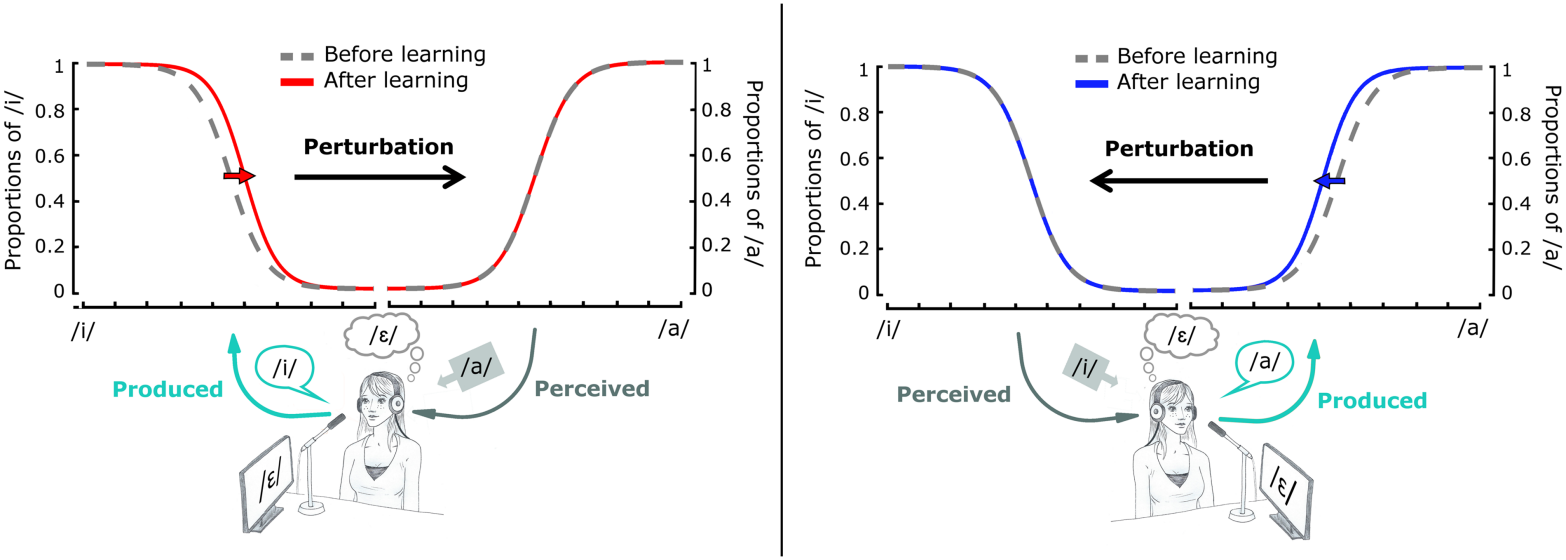
Illustration of results obtained by Lametti et al. [1]. Perceptual categorization curves before and after motor learning. Left panel: perturbation of vowel /ɛ/ toward vowel /a/. Right panel: perturbation of vowel /ɛ/ toward vowel /i/. Subjects compensate by producing sounds opposed to the direction of perturbation, closer to /i/ in the first case, and closer to /a/ in the second. Perceptual boundary shifts are observed for both directions of perturbations. The shift goes in the same direction as the perturbation, and is present only in the portion of auditory space corresponding to the productions of subjects during the compensation. Adapted from Lametti et al. [1].

Five years later, Lametti et al. [1] published a new study (referred to as “L-14” henceforth) aiming at clarifying whether the observed perceptual change was related to “the change to motor function that occurs during learning, [to the] perceptual learning related to the altered sensory inputs, [or to] some combination of the two”(p 10339). To this end they proposed an original experimental design supposed to disentangle the effects of sensory vs. motor processes on perceptual categorization. While in S-09 a perturbation of the fricative /s/ was introduced in only one direction (toward the fricative /∫/), in L-14 the vowel /ɛ/ was perturbed in two directions. For one group of subjects, the perturbation was applied toward the vowel /a/ by increasing the frequency of the first formant *F*_1_ (left panel in Fig 1). For the other group it was applied toward the vowel /i/ by decreasing *F*_1_ (right panel in Fig 1).

To make the reasoning in L-14 clear, let us analyze the case of the perturbation toward /a/ (see Fig 1, left panel). The shift of the auditory percept along the /ɛ-a/ continuum generated a compensatory movement of the tongue frontwards, which corresponds in the absence of perturbation to an auditory percept along the /ɛ-i/ continuum. Since compensation is never complete, it results with altered auditory feedback in /ɛ/ sounds that remain partly perturbed and belong to the /ɛ-a/ region, while speaker’s gestures and their corresponding somatosensory information actually belong to the /ɛ-i/ region. This is the clever method used by the authors to attempt to disentangle auditory and motor interpretations of the perturbation effects. Indeed, in their reasoning, measuring the shift of the perceptual boundary between /ɛ/ and /a/ provides a measure of the effect of the altered sensory inputs on perceptual categories, while measuring the shift of the perceptual boundary between /ɛ/ and /i/ provides a measure of the effects of the changed articulation, i.e. of the motor function, on perceptual categories. A symmetric reasoning applies for the perturbation toward /i/ (Fig 1, right panel).

Concerning motor learning, consistent with S-09 and other auditory perturbation studies in speech, motor compensation was observed and its magnitude was on average below 40% of the amplitude of perturbation. Concerning perception, a significant boundary shift was also observed in L-14. Consistent with observations reported in S-09, the resulting perceptual shifts were in the same direction as the perturbation. However, contrary to S-09, no significant shift was observed in L-14 in the region of the altered auditory inputs (i.e. the /ɛ-a/ continuum for a perturbation towards /a/); the significant shift was found in the region corresponding to the altered articulation (i.e. the /ɛ-i/ continuum for a perturbation toward /a/, see Fig 1, left panel). A control group in which subjects produced the same sequence of sounds without alteration of the auditory feedback did not show any perceptual boundary shift.

The authors concluded that their findings are “consistent with the idea that changes to central motor commands associated with speech learning are the source of changes observed in the perceptual classification of speech sounds” [1, p 10340]. Notice that if it is true that the origin of the observed perceptual shift is due to motor functions, greater changes in motor functions should induce greater changes in perception, inducing after learning positive correlations between the amount of compensation and the amplitude of the resulting perceptual shift. Intriguingly, an absence of significant correlation was reported in L-14.

### Summary of experimental results we aim at modeling

Our aim is to exploit a previously defined computational framework [12–14] modelling the interactions of perception and production in speech communication, and to apply it to model and better understand the experimental data of L-14. In our modeling approach our prime concern is to extract the deeper meaning of the experimental observations and to specify a limited number of facts that best characterize them. The following summary presents the main experimental facts on which we will focus in our modeling work.

1. **Changes in speech production induced by auditory perturbations**.
  - **Motor compensation:** speaker’s articulatory movements are modified to reduce the impact of the perturbation on the perceived sound.
  - **Incomplete compensation:** compensatory maneuvers never fully cancel the effects of the perturbation. On average, compensatory spectral changes are always below 40% of the magnitude of perturbation.
  - **Motor adaptation:** when the perturbation is removed after the learning phase, changes in speech production remain during a certain number of trials. This so called after-effect reflects a reorganization of the motor planning process that precedes motor execution of speech gestures.
2. **Changes in speech perception**. Both motor adaptation studies, S-09 and L-14, report shifts in boundaries between phonemic perceptual categories. The key-observations are:
  - **Consistency in the direction:** on average, across subjects, the direction of the shift is the same as the direction of the perturbation in both L-14 and S-09.
  - **Presence of an asymmetry:** in L-14 a significant perceptual boundary shift was observed only in the portion of the auditory space related to the articulation of subjects when compensating for the perturbation, and not in the portion of auditory space related to what subjects heard in presence of the perturbation. This asymmetry was not explored in S-09 on fricative /s/ because there is no phoneme category beyond /s/ in a direction opposite to /∫/ along the spectral continuum /s-∫/. It should be noted though that the results of S-09 tend to contradict the interpretation provided in L-14 since they describe a perceptual shift in the portion of the space related to what subjects heard in the presence of the auditory perturbation.
3. **Absence of correlations between amounts of motor compensation and perceptual shift**. Both the amount of motor compensation and the amount of perceptual boundary shift differ across subjects. While one would expect a relation between the amount of compensation and the resulting perceptual shift, no significant correlation was found in L-14.

## Model

This section introduces our model, which is an instance of the Bayesian algorithmic modeling framework [15], that is, the application of Bayesian Programming [16] to Marr’s algorithmic level of cognitive modeling [17]. With this framework, we have previously developed a series of models, under the COSMO moniker, to study speech perception and speech production in different contexts, such as speech communication and the emergence of phonological systems [13], speech perception in adverse conditions [12, 14], sensorimotor learning [18] and the emergence of speech idiosyncrasies [19]. Variants have also been applied, in speech production, to token-to-token variability [20], the incorporation of multiple constraints in speech planning [21] and the modeling of multisensory (acoustic and somatosensory) speech targets [9]. It is this last variant that we adapt here to our current study.

In the Bayesian algorithmic modeling approach, an overarching feature is that perception and production processes are not directly modeled. Instead, we build an undirected model of speech-relevant knowledge using probability distributions. Then, from this model, we compute distributions using Bayesian inference to simulate perception and production tasks. Perception and production processes, therefore, if they involve the same knowledge, become related. Let us consider the case of speech: in our approach, we commonly assume that the description of acoustic targets in speech planning is the same piece of knowledge as would be used in a purely auditory decoder in speech perception. This distinction between the knowledge stored in the model and its use to generate processes makes our framework ideal for the study of the links between production and perception mechanisms, such as those addressed in this work.

The model includes selected aspects of speech production and speech perception that are described in Section “Selected aspects for modeling”. Their implementation in the model is explained in Sections “Model definition” and “Formulation of speech production and perception questions”. The strategy used to simulate the experimental paradigm of L-14 is detailed in Section “Implementation of the experimental paradigm: Normal vs. adapted conditions”. Finally, the simulation results and their analysis are presented in Section “Results”.

### Selected aspects for modeling

Our aim is to study the interaction between speech production and speech perception processes in light of the experimental results provided in L-14. The first step in such a modeling approach consists in reducing the complexity of the experimental world into a core set of simplified components likely to capture its essential ingredients. This simplification phase should result in constraining and focusing both model implementation and results interpretation. We have selected a reduced number of aspects in speech production and speech perception that we consider to be crucial and sufficiently representative for the investigation of the interaction between motor learning and perception of isolated phonemes—here, isolated vowels /i/, /ɛ/ and /a/.

1. **Considering the stable states before and after learning**. We do not consider the particular details of the trial-to-trial evolution of the adaptation process during the training phase. Instead, we only focus on the stable states preceding and reached at the end of the adaptation process.
2. **Priority is given to speech motor planning**. We do not include any modeling of the execution of speech production gestures, ignoring in particular online feedback correction mechanisms, and only focus on the early offline planning stage preceding motor execution.
3. **Time independent states**. In the context of the two previous assumptions, we further simplify the speech production and perception systems by considering only time independent motor and sensory states that would correspond to stable vowel utterances.
4. **One-dimensional linear description**. Since both experimental designs in S-09 and L-14 studied perturbation and perception along a single dimension of the auditory space, we formally reduce the high dimensionality of motor and sensory spaces to a unique dimension. In addition, as a first order approximation, we assume that the relation between motor and sensory spaces is linear. This one-dimensional-linear simplification cannot account for the well-known many-to-one relationships between motor commands and articulatory configurations (most evident in co-contraction [22]), on the one hand, and articulatory configuration and acoustic signal on the other hand [23]. However, while this aspect would be crucial in motor learning based on articulatory perturbation (bite-block, lip-tube, jaw perturbation) requiring the use of motor-equivalence strategies for the subjects to compensate for the perturbation, it is not at the core of the mechanisms investigated in S-09 and L-14. Hence, for the sake of computational simplicity and interpretability of the results, we discard this complexity from the present analysis. This enables to take a coarse grain view and to focus on qualitative effects concerning different possible assumptions about motor adaptation, which will be introduced in Section “Implementation of the experimental paradigm: Normal vs. adapted conditions”.
5. **Auditory and somatosensory properties of the sensory representations of speech units**. Finally, a fundamental question underlying the definition of our model concerns the sensory nature of speech units. Sensory representations are usually assumed to account for classification of speech sounds in perception and for the definition of motor goals in production. Concerning production, the presence of compensatory behavior induced by auditory perturbations has been a main argument supporting the hypothesis that speech motor goals are essentially characterized in auditory terms [24, 25]. However, somatosensory perturbation studies have also reported significant compensation in speech related movement, also suggesting the existence of somatosensory characterizations of speech motor goals [26, 27]. Concerning perception, auditory representations of course play a key role. This has been confirmed for the perception of self-generated speech via perturbation experiments such as those using lip tubes or perturbation of the auditory feedback [28, 29] and it is in line with all reviews of the neuroanatomy of speech perception (e.g. [30–33]). However it remains unclear whether these are the only sensory representations that may be involved. In particular, a number of studies show an influence of somatosensory inputs on the perception of speech sounds [34] and neurocognitive data converge on the view that somatosensory regions are involved in speech processing (see a recent review by Skipper et al. [35]), suggesting a possible involvement of somatosensory representations as well. Our position with respect to these questions is the following:
  - **In production**, we assume the involvement of both auditory and somatosensory representations.
  - **In perception**, we consider two alternatives and evaluate their consequences in our framework: either perception of speech sounds involves auditory representations only or it involves both auditory and somatosensory representations. This will provide the underlying key question of this work, namely whether the data reported in L-14 do support the involvement of the speech production system in speech perception through the somatosensory system.

### Model definition

**Fig 2:**
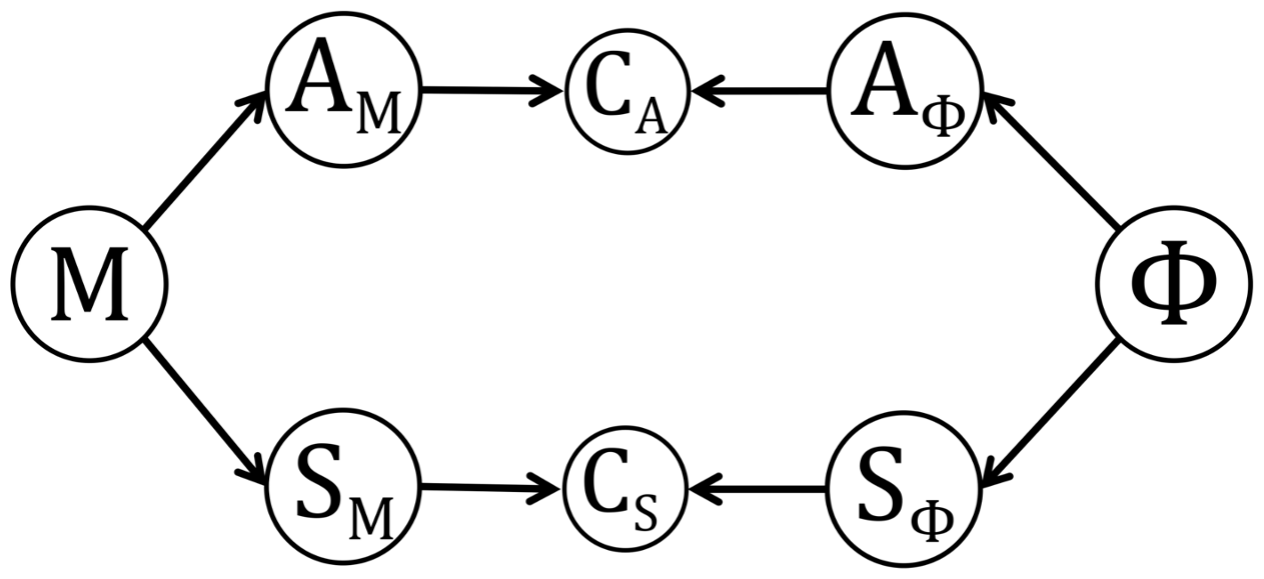
Graphical representation of model dependencies. Nodes represent variables and arrows display dependency relations. Variable Φ corresponds to phonemes, which are characterized in terms of auditory and somatosensory variables *A*_Φ_ and *S*_Φ_. Variables *A*_*M*_ and *S*_*M*_ represent the predicted auditory and somatosensory consequences of the motor commands *M*. Variables *C*_*A*_ and *C*_*S*_ implement two sensory-matching constraints that allow the connection of the corresponding sensory pathways.

The structure of the model consists in implementing a chain of probabilistic dependencies between phonological, motor and sensory variables. Variables and their dependencies are illustrated in Fig 2, and we now describe the most salient aspects of the model (a more complete mathematical description is provided in Supporting information S1 Text.

#### Variables

Variables in the model can be grouped into three sets. The first set is structured around variable *M*, which represents the set of motor commands that drive speech gestures. Associated to this variable are two “sensory-motor” variables, *A*_*M*_ and *S*_*M*_, which represent respectively the expected auditory and somatosensory consequences of motor commands *M*. As stated previously, both motor and sensory-motor variables are assumed to be one-dimensional continuous variables.

The second set is structured around variable Φ, which represents the units of speech to be produced or perceived. As stated previously, we only consider vowels /i/, /ɛ/ and /a/. Associated to variable Φ are two “sensory-phonological” variables, *A*_Φ_ and *S*_Φ_, which characterize these speech units in auditory and somatosensory terms respectively. As for sensory-motor variables, sensory-phonological variables are assumed to be one-dimensional continuous variables.

The last set of variables link sensory-phonological and sensory-motor variables. *C*_*A*_ and *C*_*S*_ are two coherence variables, which are “probabilistic connectors” between variables *A*_*M*_ and *A*_Φ_, for *C*_*A*_, and between variables *S*_*M*_ and *S*_Φ_, for *C*_*S*_. These connectors can be either left “open”, in which case the variables they link are mathematically independent, or “closed”, in which case the variables they link are forced to have the same value by a matching constraint. As such, these coherence variables can be interpreted as a “mathematical trick” to implement Bayesian switches [16, 36] controlling the propagation of information in the model.

#### Dependencies: Decomposition of the joint probability distribution

The joint probability distribution is decomposed as a product of elementary terms:

$$\begin{array}{c} P(M\;S_{M}\;A_{M}\;\Phi \;S_{\Phi }\;A_{\Phi }\;C_{S}\;C_{A})\\ =P\left(M\right)P\left(A_{M}\;|\;M\right)P\left(S_{M}\;|\;M\right)\\ P\left(\Phi \right)P\left(A_{\Phi }\;|\;\Phi \right)P\left(S_{\Phi }\;|\;\Phi \right)\\ P\left(C_{A}\;|\;A_{M}\;A_{\Phi }\right)P\left(C_{S}\;|\;S_{M}\;S_{\Phi }\right). \end{array}$$ (1)

This decomposition, illustrated in Fig 2, relies on a certain number of conditional independence hypotheses that we do not discuss here (but see Supporting information S1 Text for details).

#### Parametric forms

We now define each probability distribution of Eq (1). Concerning prior distributions *P*(*M*) and *P*(Φ), we assume no prior knowledge concerning values of variables *M* and Φ. Therefore, we identify *P*(*M*) and *P*(Φ) with uniform distributions.

*P*(*A*_*M*_ | *M*) and *P*(*S*_*M*_ | *M*) represent knowledge relating motor commands to their predicted sensory consequences. They correspond to sensory-motor internal forward models often assumed to be involved in motor planning [37–39] (but see [40, 41] for debates). As explained in Section “Selected aspects for modeling”, for the sake of computational simplicity, we assume that these stored relations are linear. The corresponding auditory-motor and somatosensory-motor mappings, *ρ*_*A*_(*m*) and *ρ*_*S*_(*m*), are defined as follows:

$$\begin{array}{ccc} \rho_{A}\left(m\right) & ≔ & \alpha_{A}.m+\beta_{A}, \end{array}$$ (2)

$$\begin{array}{ccc} \rho_{S}\left(m\right) & ≔ & \alpha_{S}.m+\beta_{S}, \end{array}$$ (3)

where values of parameters *α*_*A*_, *α*_*S*_, *β*_*A*_ and *β*_*S*_ depend on further hypotheses that will be specified in Section “Implementation of the experimental paradigm: Normal vs. adapted conditions”. Finally, we further assume that the stored sensory-motor internal models have infinite precision and are therefore deterministic, such that *P*(*A*_*M*_ | *M*) and *P*(*S*_*M*_ | *M*) are identified with Dirac delta functions:

$$\begin{array}{ccc} P(\left[A_{M}=a\right]\;|\;\left[M=m\right]) & ≔ & \delta (a-\rho_{A}\left(m\right)), \end{array}$$ (4)

$$\begin{array}{ccc} P(\left[S_{M}=s\right]\;|\;\left[M=m\right]) & ≔ & \delta (s-\rho_{S}\left(m\right)). \end{array}$$ (5)

*P*(*A*_Φ_ | Φ) and *P*(*S*_Φ_ | Φ) correspond to the auditory and somatosensory characterizations of phonemes. As it is common in other modeling studies [42–46], we identify them with Gaussian distributions specified by their means and standard-deviations $\left(\mu_{A}^{\phi },\sigma_{A}^{\phi }\right)$ and $\left(\mu_{S}^{\phi },\sigma_{S}^{\phi }\right)$ for each phoneme *ϕ* in auditory and somatosensory terms. Values of parameters $\left(\mu_{A}^{\phi },\sigma_{A}^{\phi }\right)$ and $\left(\mu_{S}^{\phi },\sigma_{S}^{\phi }\right)$ depend on further hypotheses and will be specified in Section “Implementation of the experimental paradigm: Normal vs. adapted conditions” and “Update of the auditory-motor internal model *P*(*A*_*M*_ | *M*)”.

*P*(*C*_*A*_ | *A*_*M*_ *A*_Φ_) and *P*(*C*_*S*_ | *S*_*M*_ *S*_Φ_) implement the sensory matching constraints relating sensory-motor and sensory-phonological variables in the following way:

$$\begin{array}{ccc} P(\left[C_{A}=1\right]\;|\;\left[A_{M}=a_{m}\right]\;\left[A_{\Phi }=a_{\phi }\right]) & ≔ & \left\{ \begin{array}{cc} 1 & \text{if}\;a_{m}=a_{\phi }\\ 0 & \text{otherwise} \end{array}\right. \end{array}$$ (6)

$$\begin{array}{ccc} P(\left[C_{S}=1\right]\;|\;\left[S_{M}=s_{m}\right]\;\left[S_{\Phi }=s_{\phi }\right]) & ≔ & \left\{ \begin{array}{cc} 1 & \text{if}\;s_{m}=s_{\phi }\\ 0 & \text{otherwise} \end{array}\right. \end{array}$$ (7)

### Formulation of speech production and perception questions

In the previous section we proposed a computational definition of the joint probability distribution of the model. This definition was based on particular assumptions concerning relations between variables. The Bayesian formalism allows to simulate speech production and perception by defining and computing probability distributions of interest, that we call “questions”.

#### Speech production questions

Speech production questions correspond to the inference of motor commands for the production of a desired phoneme. The dependence structure of Fig 2 shows that if coherence variables *C*_*A*_ and *C*_*S*_ are not assumed to be 1, that is to say, if they are “Bayesian switches” left open, there is no dependency between *M* and Φ, which would correspond to an unrealistic situation (see Supporting information S2 Text for further details). Instead, inferring motor commands for the production of a given phoneme with either variable *C*_*A*_ or variable *C*_*S*_ or both set to 1, leads to three planning processes that can be characterized as follows.

1. The first planning process is based on the auditory pathway only and corresponds to:
  $$\begin{array}{c} P([M=m]\;|\;\Phi \;[C_{A}=1])\\ \propto P([A_{\Phi }=\rho_{A}(m)]\;|\;\Phi ). \end{array}$$ (8)
2. The second planning process is based on the somatosensory pathway only and corresponds to:
  $$\begin{array}{c} P([M=m]\;|\;\Phi \;[C_{S}=1])\\ \propto P([S_{\Phi }=\rho_{S}(m)]\;|\;\Phi ). \end{array}$$ (9)
3. The third planning process is based on the fusion of auditory and somatosensory pathways and corresponds to:
  $$\begin{array}{l} P([M=m]\;|\;\Phi \;[C_{A}=1]\;[C_{S}=1])\\ \propto P([A_{\Phi }=\rho_{A}(m)]\;|\;\Phi )P([S_{\Phi }=\rho_{S}(m)]\;|\;\Phi ), \end{array}$$ (10)

These equations are obtained by the application of Bayesian inference rules to the joint probability distribution given by Eq (1). Derivations are provided in Supporting information S2 Text. All terms on the right hand sides of Eqs (8), (9) and (10) were defined in Section “Parametric forms”.

The probability of selecting a particular motor command *m* is hence proportional to the probability that the predicted sensory consequences of *m* (expressed by *ρ*_*A*_(*m*) and *ρ*_*S*_(*m*) in auditory and somatsoensory terms) are in agreement with the sensory characterization of the intended phoneme in the corresponding sensory pathway.

#### Speech perception questions

Perception questions correspond to the categorization of auditory inputs into phoneme identity. We consider that the perceived auditory stimulus is a value of the auditory-motor variable *A*_*M*_. Similar to the previous production questions, we can define three categorization questions depending on the activation of variables *C*_*A*_ or *C*_*S*_.

1. The assumption that categorization is based only on the auditory pathway (as in auditory theories of speech perception) corresponds to:
  $$\begin{array}{c} P(\left[\Phi =\phi \right]\;|\;\left[A_{M}=a\right]\;\left[C_{A}=1\right])\\ =\frac{P(\left[A_{\Phi }=a\right]\;|\;\left[\Phi =\phi \right])}{\sum_{\phi^{\prime }}P\left(\left[A_{\Phi }=a\right]\;|\;\left[\Phi =\phi^{\prime }\right]\right)} \end{array}$$ (11)
2. The assumption that categorization is based only on the somatosensory pathway (as in the direct realist theory [47]) corresponds to:
  $$\begin{array}{c} P(\left[\Phi =\phi \right]\;|\;\left[A_{M}=a\right]\;\left[C_{S}=1\right])\\ =\frac{P(\left[S_{\Phi }=\rho_{S}\circ \rho_{A}^{-1}\left(a\right)\right]\;|\;\left[\Phi =\phi \right])}{\sum_{\phi^{\prime }}P\left(\left[S_{\Phi }=\rho_{S}\circ \rho_{A}^{-1}\left(a\right)\right]\;|\;\left[\Phi =\phi^{\prime }\right]\right)} \end{array}$$ (12)
3. The assumption that categorization is based on the fusion of both auditory and somatosensory pathways (as in perceptuo-motor theories [8]) corresponds to:
  $$\begin{array}{c} P(\left[\Phi =\phi \right]\;|\;\left[A_{M}=a\right]\;\left[C_{S}=1\right]\;\left[C_{A}=1\right])\\ =\frac{P\left(\left[A_{\Phi }=a\right]\;|\;\left[\Phi =\phi \right]\right)\;P\left(\left[S_{\Phi }=\rho_{S}\circ \rho_{A}^{-1}\left(a\right)\right]\;|\;\left[\Phi =\phi \right]\right)}{\sum_{\phi^{\prime }}P\left(\left[A_{\Phi }=a\right]\;|\;\left[\Phi =\phi^{\prime }\right]\right)\;P\left(\left[S_{\Phi }=\rho_{S}\circ \rho_{A}^{-1}\left(a\right)\right]\;|\;\left[\Phi =\phi^{\prime }\right]\right)}. \end{array}$$ (13)

The symbol ∘ in Eqs (12) and (13) denotes the composition operator, and therefore $\rho_{S}\circ \rho_{A}^{-1}\left(a\right)$ corresponds to the somatosensory image of the auditory value *a* as obtained first by the identification of motor commands *m* achieving the production of *a* ($m=\rho_{A}^{-1}\left(a\right)$) and then by the prediction of the somatosensory variable *s* generated from the inferred motor commands (*s* = *ρ*_*S*_(*m*)). Solutions for these three inference questions are obtained from the joint probability distribution given by Eq (1). Details of the derivation are provided in Supporting information S2 Text.

These equations express the way Bayesian computation yields categorization processes from the structure and knowledge encoded in the model. Under the auditory pathway case, the probability of categorizing an auditory input *a* into phoneme *ϕ* is obtained by evaluating the probability that this auditory input would correspond to the auditory characterization of the considered phoneme (*P*([*A*_Φ_ = *a*] | [Φ = *ϕ*]) in the numerator), and comparing it to the probability that it would correspond to the auditory characterization of any of the possible phonemes (the sum over *ϕ*′ on the denominator). When this ratio is close to 1, the auditory value is categorized as phoneme *ϕ* with full certainty. The smaller the ratio, the lower the probability of this categorization.

Consider, for instance, the case of the categorization of an auditory input *a* into the phoneme /i/ (among the three vowels /i, ɛ, a/ in our example) as given by Eq (11). Replacing *P*(*A*_Φ_ | Φ) with their definition as Gaussian probability distributions yields:

$$\begin{array}{c} P(\left[\Phi =/\text{i}/\right]\;|\;\left[A_{M}=a\right]\;\left[C_{A}=1\right])\\ =\frac{e^{-\frac{{\left(a-\mu_{A}^{i}\right)}^{2}}{2{\sigma_{A}^{i}}^{2}}}}{e^{-\frac{{\left(a-\mu_{A}^{i}\right)}^{2}}{2{\sigma_{A}^{i}}^{2}}}+e^{-\frac{{\left(a-\mu_{A}^{\epsilon }\right)}^{2}}{2{\sigma_{A}^{\epsilon }}^{2}}}+e^{-\frac{{\left(a-\mu_{A}^{a}\right)}^{2}}{2{\sigma_{A}^{a}}^{2}}}}. \end{array}$$ (14)

**Fig 3:**
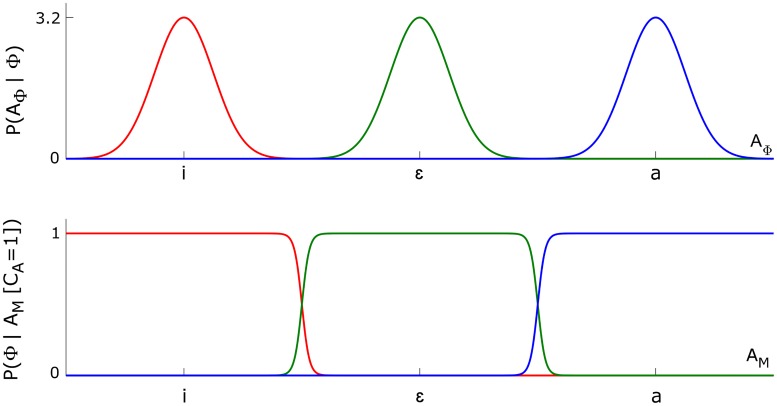
Auditory characterizations and corresponding phoneme categorization functions. Top panel: auditory characterization, *P*(*A*_Φ_ | Φ), for phoneme /i/ (red), phoneme /ɛ/ (green) and phoneme /a/ (blue). Bottom panel: categorization functions under the auditory pathway approach, *P*(Φ | *A*_*M*_ [*C*_*A*_ = 1]), obtained from auditory characterizations according to Eq (11). Eq (14) gives the explicit form for phoneme /i/.

This function is illustrated in Fig 3 for parameter values in the normal condition, as specified in Section “Normal condition: Initial values of parameters”. The corresponding categorization functions under the somatosensory and fusion of pathways are derived essentially in the same way.

#### Selection of production and perception questions

We have derived 3 perception and 3 production questions that differ with respect to the sensory pathways assumed to be involved in these processes. For the sake of brevity, we limit the presentation of simulations and do not consider the outcome of all of the 9 combinations of questions, in order to focus on those that correspond to the richest scientific contributions. Therefore, as pointed out in Section “Selected aspects for modeling”, concerning production we consider only the question assuming the fusion of auditory and somatosensory pathways, *P*(*M* | Φ [*C*_*A*_ = 1] [*C*_*S*_ = 1]). Concerning perception we keep and compare questions assuming the involvement of the auditory pathway alone, *P*(Φ | *A*_*M*_ [*C*_*A*_ = 1]), and the fusion of sensory pathways, *P*(Φ | *A*_*M*_ [*C*_*A*_ = 1] [*C*_*S*_ = 1]).

In order to simplify notations, we denote the selected production and perception questions by:

$$\begin{array}{ccc} Q_{\text{Prod}}^{F} & ≔ & P(M\;|\;\Phi \;\left[C_{A}=1\right]\;\left[C_{S}=1\right]),\\ Q_{\text{Per}}^{A} & ≔ & P(\Phi \;|\;A_{M}\;\left[C_{A}=1\right]),\\ Q_{\text{Per}}^{F} & ≔ & P(\Phi \;|\;A_{M}\;\left[C_{A}=1\right]\;\left[C_{S}=1\right]). \end{array}$$

### Implementation of the experimental paradigm: Normal vs. adapted conditions

Our aim is to simulate and compare the outcome of the production and perception tests in L-14, prior to the auditory perturbation and after the training phase, i.e. when perturbation is removed and adaptation has been reached. These tests are naturally implemented in the model as the outcome of the production and perception questions defined in the previous section.

Adaptation is implemented as the update of a part of the knowledge included in the model. This knowledge is represented by the four relations defined in Section “Parametric forms”: the two sensory-motor internal models, *P*(*A*_*M*_ | *M*) and *P*(*S*_*M*_ | *M*), and the two sensory characterizations of phonemes, *P*(*A*_Φ_ | Φ) and *P*(*S*_Φ_ | Φ). In this context, normal and adapted conditions are implemented by different values of the parameters characterizing these relations. Values of parameters in normal condition are arbitrary initial values. This is why we chose them to be as simple as possible. They are specified in Section “Normal condition: Initial values of parameters”.

Two fundamental questions remain to be answered in order to specify how adaptation will affect these initial values: (1) which of the four relations is changed during adaptation, and (2) how? The first question actually rephrases in computational terms the question raised in L-14 (p 10339), and quoted in its original formulation in Section “Influence of motor learning upon speech perception: overview of experimental facts”, extending it to behavioral changes in both production and perception: “So what produces the [behavioral changes] during motor learning? Is it the change to [parameters of the sensory-motor internal models], that occurs during learning? Is it changes to [parameters of the sensory characterizations of phonemes], related to the altered sensory inputs? Or is it some combination of the two?”.

In the following sections, we address these two questions in two steps. In Section “Adaptation hypotheses” we partially answer the first question by motivating the selection of a subset of possible changes induced by adaptation. In Section “Results” we further answer these questions by evaluating the outcome of different implementations of the selected changes and by comparing them with the experimental facts summarized in Section “Summary of experimental results we aim at modeling”.

#### Normal condition: Initial values of parameters

We now specify parameters of the two sensory-motor internal models, *P*(*A*_*M*_ | *M*) and *P*(*S*_*M*_ | *M*), and the two sensory characterizations of phonemes, *P*(*A*_Φ_ | Φ) and *P*(*S*_Φ_ | Φ).

The two sensory-motor internal models are defined in terms of auditory-motor and somatosensory-motor mappings *ρ*_*A*_ and *ρ*_*S*_, which are characterized by parameters *α*_*A*_, *β*_*A*_ and *α*_*S*_, *β*_*S*_. Without loss of generality, we define metric units of motor and sensory spaces in order to have *α*_*A*_ = *α*_*S*_ = 1 and *β*_*A*_ = *β*_*S*_ = 0 in normal condition. Therefore, with $\rho_{A}^{n}$ and $\rho_{S}^{n}$ being the auditory-motor and somatosensory-motor mappings in normal conditions respectively, we have:

$$\begin{array}{ccc} \rho_{A}^{\left(n\right)}\left(m\right) & = & m, \end{array}$$ (15)

$$\begin{array}{ccc} \rho_{S}^{\left(n\right)}\left(m\right) & = & m. \end{array}$$ (16)

**Fig 4:**
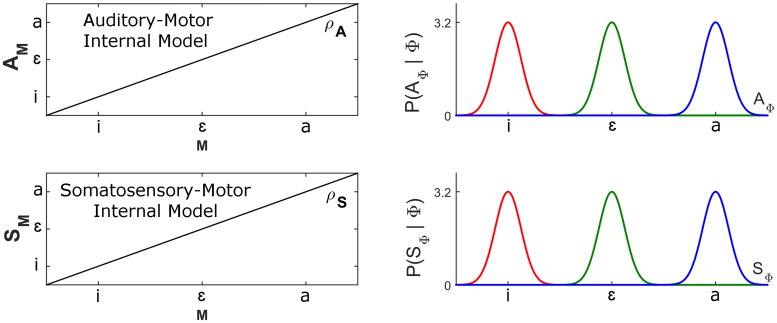
Stored sensory-motor mappings and sensory characterizations of phonemes under normal conditions. Left panels: auditory-motor internal mapping $\rho_{A}^{n}$ (top) and somatosensory-motor internal mapping $\rho_{S}^{n}$ (bottom). Both mappings are assumed to be identity. Right panels: auditory (top) and somatosensory (bottom) characterization of phonemes. Probability distributions are all Gaussian, evenly distributed in each space, and with equal standard-deviations, equal to $\frac{1}{8}$ of the distance between phonemes.

The left panels of Fig 4 illustrate these sensory-motor mappings.

The two sensory characterizations of phonemes are defined as Gaussian probability distributions with means and standard-deviations, $\left(\mu_{A}^{\phi },\sigma_{A}^{\phi }\right)$ and $\left(\mu_{S}^{\phi },\sigma_{S}^{\phi }\right)$.

For the sake of simplicity, we assume that in normal condition these sensory characterizations are evenly distributed in both sensory spaces with the same standard-deviations equal to $\frac{1}{8}$ of the distance between neighboring phonemes (see Supporting information S4 Text for further details). The right panels of Fig 4 illustrate the corresponding probability distributions.

**Fig 5:**
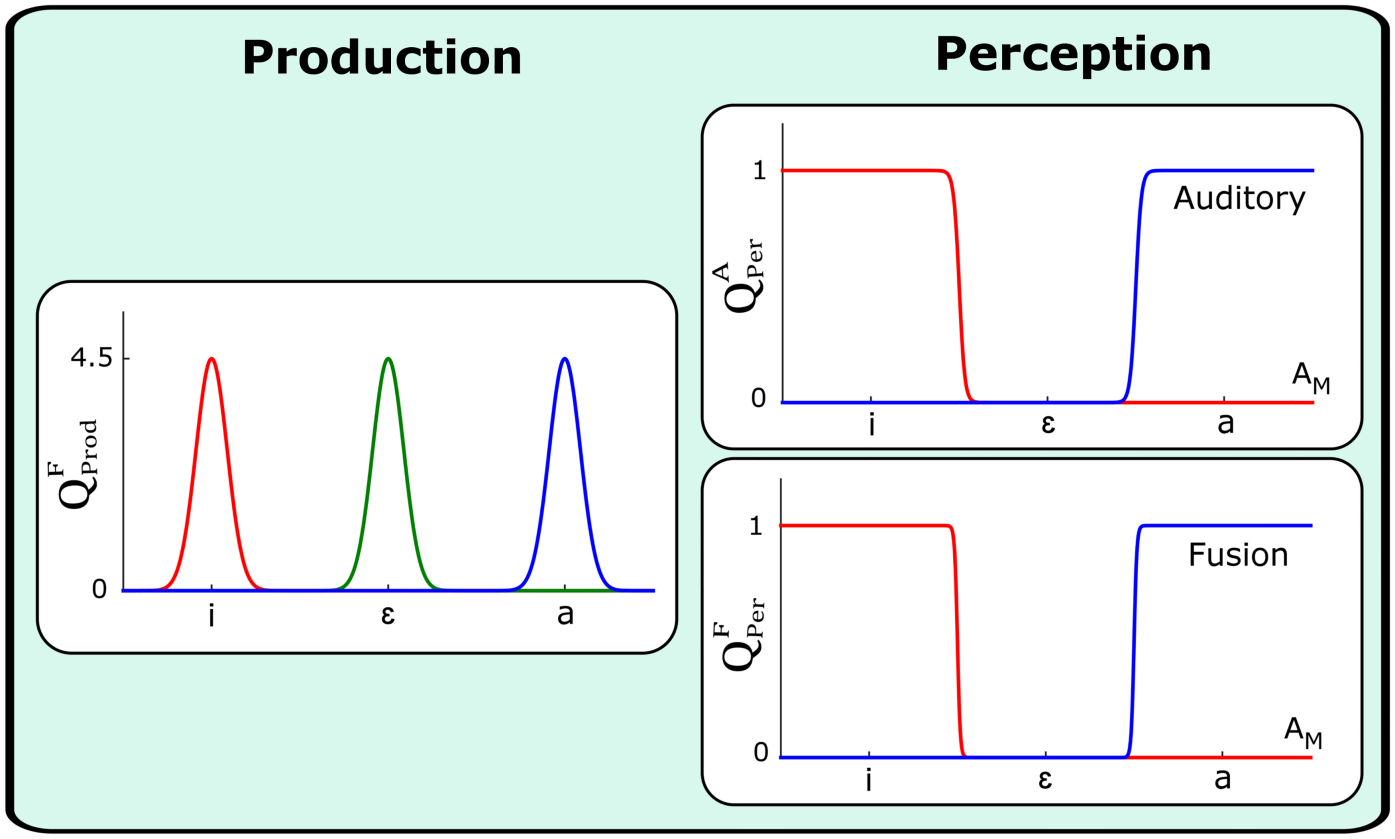
Outcome of the production question (left panel) and perception questions (right panels) under normal conditions. The categorization function corresponding to vowel /ɛ/ is not represented for clarity of the figure and since it corresponds to the complementary of the two other curves (as it can be seen in the bottom panel of Fig 3).

Since the model is now completely defined in normal conditions, we can study the outcome of the production and perception questions, which correspond to production and perception pretests in L-14. The corresponding functions are displayed in Fig 5.

Concerning production, Eq (10) indicates that the outcome of the planning process is a product of two Gaussian probability distributions. The product is known to result into a new Gaussian probability distribution with smaller variance [48], as it can be seen in the left panel displayed in Fig 5.

Concerning perception, the outcome of the two perception processes corresponds to categorization functions with the same positions of the boundaries, but with boundary slopes that are different. In this context, the fusion of sensory pathways results in a steeper slope than the auditory pathway alone.

#### Adaptation hypotheses

We focus now on the adapted state. Which of the two sensory-motor internal models, *P*(*A*_*M*_ | *M*) and *P*(*S*_*M*_ | *M*), or the two sensory characterizations of phonemes, *P*(*A*_Φ_ | Φ) and *P*(*S*_Φ_ | Φ) is being updated during the training phase? We consider that any of these relations may be updated if the perturbation introduced during the training phase leads to considering that they are no longer correct.

Since the perturbation of the auditory feedback only affects the relation between motor commands and auditory outputs, we do not introduce any change to the somatosensory-motor internal model, *P*(*S*_*M*_ | *M*), but we assume that the auditory-motor internal model, *P*(*A*_*M*_ | *M*), may be updated in order to learn the new auditory-motor relation.

The auditory perturbation also induces a mismatch between the perturbed auditory output and the learned phoneme characterization *P*(*A*_Φ_ | Φ). This mismatch can be resolved by an update of the auditory-motor internal model alone, *P*(*A*_*M*_ | *M*), so that under the perturbed condition new motor commands are associated to the usual auditory region characterizing the produced phoneme. However, modifying the auditory characterization of phonemes, *P*(*A*_Φ_ | Φ), may also contribute to the reduction of this mismatch. In S-09 the reported results were interpreted as a combination of these two hypotheses. The authors suggested that “speech adaptation to altered auditory feedback is not limited to the motor domain, but rather involves changes in both motor output and auditory representations of speech sounds that together act to reduce the impact of the perturbation” [2, p 1103, abstract]. In other words, subjects could reduce the impact of the perturbation by changing the motor commands associated to the production of the phoneme, but also by modifying their stored auditory characterizations of speech sounds. Furthermore, as in L-14 we only focus on the perturbation of vowel /ɛ/. Hence, among the stored auditory characterizations we consider that only *P*(*A*_Φ_ | [Φ = /ɛ/]), corresponding to vowel /ɛ/, may be updated.

The motor command change resulting from the compensation for the auditory perturbation also induces a somatosensory mismatch. Indeed, somatosensory values resulting from compensation deviate from the stored somatosensory characterization of the intended phoneme. Therefore, once the compensation for the auditory perturbation starts to be efficient, the stored somatosensory characterization of the intended phoneme, *P*(*S*_Φ_ | Φ), may also change in order to match the new somatosensory patterns associated with the modified articulation. Furthermore, since we only focus on the perturbation of vowel /ɛ/, we consider that among the stored somatosensory characterizations only *P*(*S*_Φ_ | [Φ = /ɛ/]) may be updated.

In summary, we retain three possible changes that may be induced by motor adaptation:

1. An update of the auditory-motor internal model *P*(*A*_*M*_ | *M*);
2. An update of the auditory characterization of the perturbed vowel *P*(*A*_Φ_ | [Φ = /ɛ/]);
3. An update of the somatosensory characterization of the perturbed vowel *P*(*S*_Φ_ | [Φ = /ɛ/]).

These three possible changes result in 7 possible adaptation hypotheses, depending on whether we combine one, two or the three of them.

Section “Results” aims to evaluate which of these adaptation hypotheses may account for the experimental facts reported in L-14. This evaluation is performed by comparing the consequences of each hypothesis with respect to compensation and perceptual boundary shift as reported in Section “Summary of experimental results we aim at modeling”.

We assess the direction and amount of compensation via the displacement of the motor planning distribution $Q_{\text{Prod}}^{F}$ associated with /ɛ/ in the motor command space. We evaluate the amount of perceptual boundary shift via the displacement of the point where the categorization function $Q_{\text{Per}}^{A}$ or $Q_{\text{Per}}^{F}$ takes value $\frac{1}{2}$.

Finally, since the behavior of the model is symmetric around vowel /ɛ/, we focus only on the case of a perturbation in the direction of vowel /a/ (left panel of Fig 1). All simulations are therefore performed assuming a perturbation with a magnitude of 40% of the distance between neighboring phonemes and in the direction of vowel /a/.

## Results

The primary goal of this section is to evaluate which of the 7 adaptation hypotheses account for the experimental facts reported in L-14. To do so, we proceed sequentially: we first focus on perception and evaluate results corresponding to the two categorization questions $Q_{\text{Per}}^{A}$ and $Q_{\text{Per}}^{F}$. For the hypotheses that are compatible with the perceptual boundary shift observed in L-14, the associated compensation in production is evaluated, and again only the hypotheses that are compatible with the results of L-14 are kept. Finally, in a third step, we further evaluate the selected adaptation hypotheses with respect to the corresponding correlations between the amount of compensation in production and the magnitude of perceptual boundary shift.

### Evaluation with respect to perception

#### Update of the auditory-motor internal model *P*(*A*_*M*_ | *M*)

**Fig 6:**
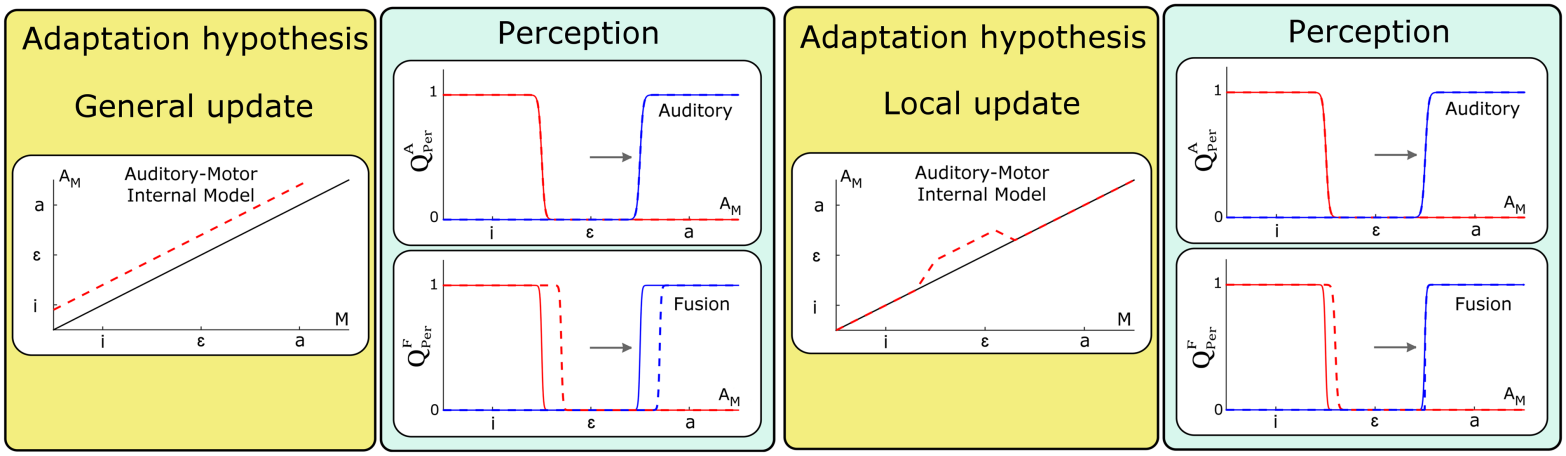
Changes in perception questions resulting from updates of the auditory-motor internal model *P*(*A*_*M*_ | *M*). Left panels: general update. Right panels: local update. Yellow panels represent the auditory-motor mapping before (solid lines) and after update (dashed lines). The magnitude and direction of update is equal to the assumed perturbation magnitude. Green panels represent the outcome of the two perception questions in normal (solid lines) vs. adapted conditions (dashed lines). Only categorization functions for vowel /i/ (red plots) and /a/ (blue plots) are displayed (categorization function for vowel /ɛ/ being complementary to the two others). The direction and magnitude of the perturbation is indicated by the horizontal arrow.

We begin by considering the consequences of an update of the auditory-motor internal forward model *P*(*A*_*M*_ | *M*) (see Fig 6) characterized by the mapping *ρ*_*A*_(*m*) and parameters *α*_*A*_ and *β*_*A*_ (see Eq (2)).

Since the perturbation corresponds to a constant shift *δ*_*A*_ in auditory space, a straightforward update of the auditory-motor mapping induced by training under the perturbed condition, $\rho_{A}^{\left(u\right)}$, can be obtained from the mapping in normal condition, $\rho_{A}^{\left(n\right)}$, as:

$$\begin{array}{c} \rho_{A}^{\left(u\right)}\left(m\right)=\rho_{A}^{\left(n\right)}\left(m\right)+\delta_{A}=m+\delta_{A}, \end{array}$$ (17)

that is to say, parameter *α*_*A*_ in Eq (2) remains unchanged (value 1) and *β*_*A*_ is updated by the amount of shift *δ*_*A*_.

This first implementation corresponds to a general update of the internal model, which is not limited to the domain of variation of the motor commands experienced by the subject during the perturbation experiment. Assuming such a generalization is a strong hypothesis that has been questioned in different experimental studies (including in speech [49, 50], and in arm movements [51]). Consequently, we also consider a second, more local, update of the internal model that is limited to the range of motor commands experienced by the subject when speaking with the perturbation. We will compare the predictions of these two assumptions on the two perception questions.

We begin by considering the general update hypothesis. The left panels of Fig 6 present the outcome for the perception questions, under the auditory pathway hypothesis $Q_{\text{Per}}^{A}$ and under the fusion of sensory pathways hypothesis $Q_{\text{Per}}^{F}$, assuming the general update of the auditory-motor internal model *P*(*A*_*M*_ | *M*). We firstly observe that updating the auditory-motor internal model results in no change in the categorization process under the auditory pathway $Q_{\text{Per}}^{A}$, consistent with Eq (11) in which only *P*(*A*_Φ_ | Φ) is involved.

In addition, we observe a perceptual boundary shift under the fusion of pathways $Q_{\text{Per}}^{F}$. This is consistent with Eq (13) where the auditory categorization under the fusion of sensory pathways involves the inverse of the auditory-motor mapping, $\rho_{A}^{-1}$, that has been updated. Importantly, it should be noted that the direction of perceptual boundary shifts (from the solid to the dotted line) is the same as the direction of the perturbation (horizontal arrow). This is consistent with the findings reported in S-09 and L-14. However, we notice that boundary shifts are present on both sides of vowel /ɛ/ in the auditory space, contrary to the asymmetry reported in L-14.

Let us consider now the local update hypothesis. The right panels of Fig 6 present the outcome for the perception questions, $Q_{\text{Per}}^{A}$ and $Q_{\text{Per}}^{F}$, assuming a local update of the auditory-motor internal model *P*(*A*_*M*_ | *M*). Details about the specification of this local update are provided in Supporting information S3 Text.

The main results are consistent with those of the general update, except that the perceptual boundary shift observed under the fusion of pathways is now restricted to the region of the auditory space associated with the interval of the motor commands space where the internal model was updated, i.e. in the domain located between /i/ and /ɛ/. The resulting asymmetry is in agreement with the observations reported by L-14. However, it is important to specify that the magnitude of the shift as well as the characteristics of the asymmetry are sensitive to the choice of the parameters determining the local update of the internal model. Here, parameters implement the hypothesis that learning is limited to a portion of motor space consistent with what subjects may have explored when speaking with the perturbation.

#### Update of the auditory characterization *P*(*A*_Φ_ | Φ)

The auditory characterization of phonemes, *P*(*A*_Φ_ | Φ), was identified, in Section “Parametric forms”, with Gaussian distributions with parameters $\left(\mu_{A}^{\phi },\sigma_{A}^{\phi }\right)$, where *ϕ* indicates the considered phoneme. In the present case, we are interested in the auditory characterization of phoneme /ɛ/, that is, in the Gaussian distribution *P*(*A*_Φ_ | [Φ = /ɛ/]) with parameters $\left(\mu_{A}^{ɛ},\sigma_{A}^{ɛ}\right)$. We consider adaptation to the auditory perturbation that moves /ɛ/ toward /a/ and assume that it may update either $\mu_{A}^{ɛ}$ or $\sigma_{A}^{ɛ}$ or both. Updating $\mu_{A}^{ɛ}$ modifies the location of the Gaussian, whereas updating $\sigma_{A}^{ɛ}$ modifies its width. We will first evaluate the effect induced by an update of each parameter independently, and then consider a combined update.

**Fig 7:**
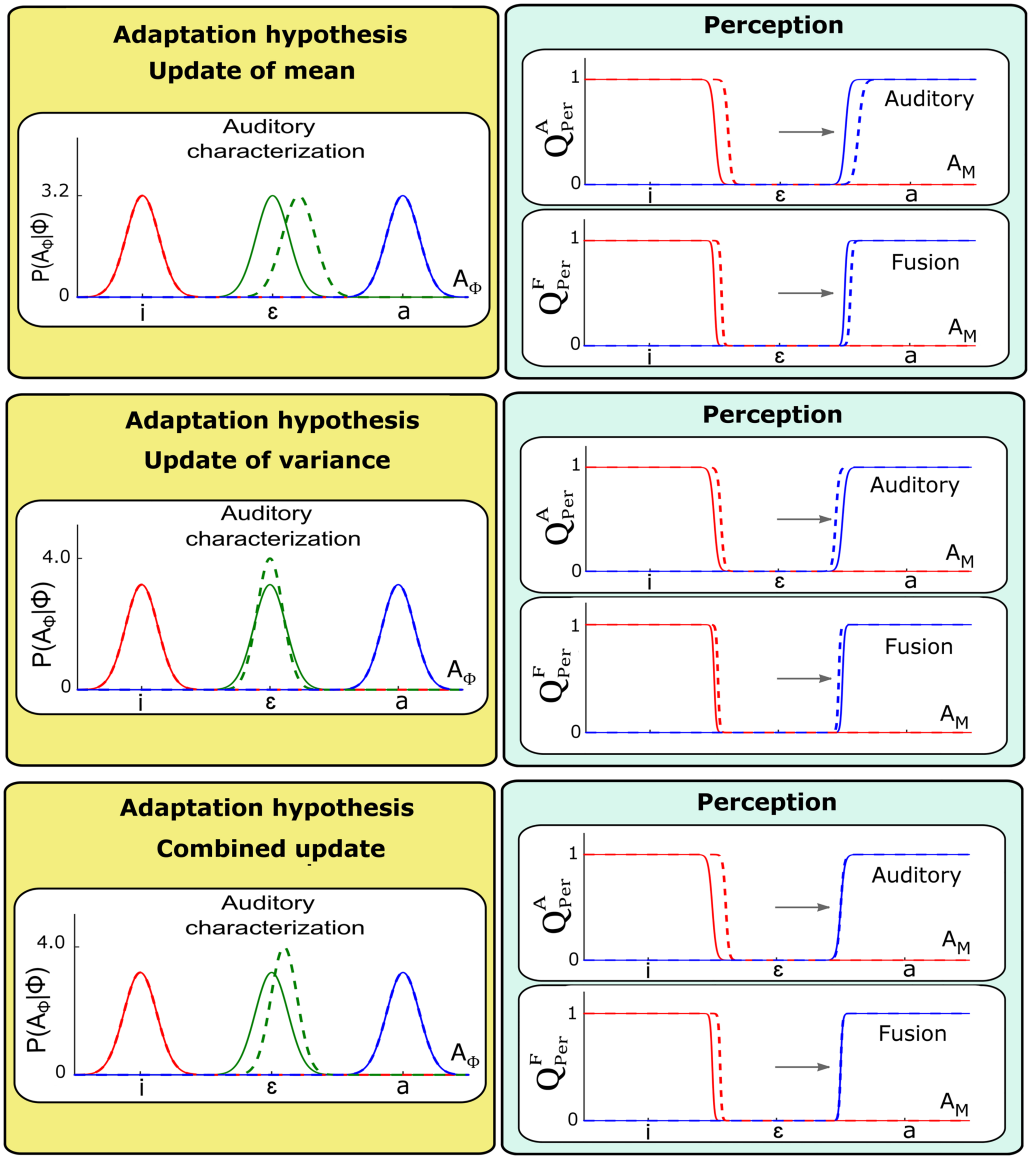
Changes in perception questions resulting from different updates of the auditory characterization of the perturbed vowel, *P*(*A*_Φ_ | [Φ = /ɛ/]). Top and middle panels correspond to independent updates of the mean $\mu_{A}^{ɛ}$ and the standard deviation $\sigma_{A}^{ɛ}$ respectively. Bottom panels correspond to a combined shift of $\mu_{A}^{ɛ}$ and reduction of $\sigma_{A}^{ɛ}$. The outcome of simulations before update (solid lines) are superimposed to those after update (dashed lines).

The top panel of Fig 7 present the outcome of the two perception questions $Q_{\text{Per}}^{A}$ and $Q_{\text{Per}}^{F}$ after a shift of $\mu_{A}^{ɛ}$ in the direction of vowel /a/. The middle panels of Fig 7 illustrate the outcome of the two perception questions resulting from a reduction of $\sigma_{A}^{ɛ}$.

We observe that modifying parameters $\mu_{A}^{ɛ}$ and $\sigma_{A}^{ɛ}$ induces changes in auditory categorization both with the auditory pathway only $Q_{\text{Per}}^{A}$ and with the fusion of sensory pathways $Q_{\text{Per}}^{F}$. However, the perceptual changes vary according to the parameter that is modified. Shifting parameter $\mu_{A}^{ɛ}$ (top panel) induces a shift of *P*(*A*_Φ_ | [Φ = /ɛ/]), resulting in a boundary shift that is similar on both sides of /ɛ/ along the auditory continuum and goes in the same direction as the shift in location of *P*(*A*_Φ_ | [Φ = /ɛ/]). Reducing parameter $\sigma_{A}^{ɛ}$ of *P*(*A*_Φ_ | [Φ = /ɛ/]) (middle panel) induces boundary shifts that are in opposite direction on both sides of /ɛ/ along the auditory continuum. The boundaries follow the narrowing of *P*(*A*_Φ_ | [Φ = /ɛ/]) on both sides, and get closer to the center of the Gaussian distribution characterizing /ɛ/.

Therefore, it appears that an adequate combination of $\mu_{A}^{ɛ}$ and $\sigma_{A}^{ɛ}$ modifications may produce a pattern in agreement with the one observed by L-14, with a boundary shift in the direction of the perturbation, obtained just on the /ɛ/-/i/ side but not on the /ɛ/-/a/ side (see Fig 7, bottom panel). The relation between $\mu_{A}^{ɛ}$ and $\sigma_{A}^{ɛ}$ implemented in the simulations of Fig 7 is provided in Supporting information S4 Text. Note that this relation has been specifically designed in order to reproduce the desired asymmetrical boundary shift, but we attach no claim of cognitive plausibility to this specific relation. We will discuss the theoretical implication of this ad-hoc choice in Section “Discussion”.

#### Update of the somatosensory characterization *P*(*S*_Φ_ | Φ)

The somatosensory characterization of phonemes *P*(*S*_Φ_ | Φ) was identified with Gaussian distributions parameterized by $\left(\mu_{S}^{\phi },\sigma_{S}^{\phi }\right)$. The articulatory changes enabling compensation for the perturbation during adaptation could generate an update of the somatosensory characterization of the produced phoneme in order to account for the somatosensory correlates corresponding to the new articulatory postures. For an auditory perturbation of vowel /ɛ/ toward /a/, the compensatory behavior leads to articulatory configurations closer to /i/. One would therefore expect a change of $\mu_{S}^{ɛ}$ in the direction of phoneme /i/.

**Fig 8:**
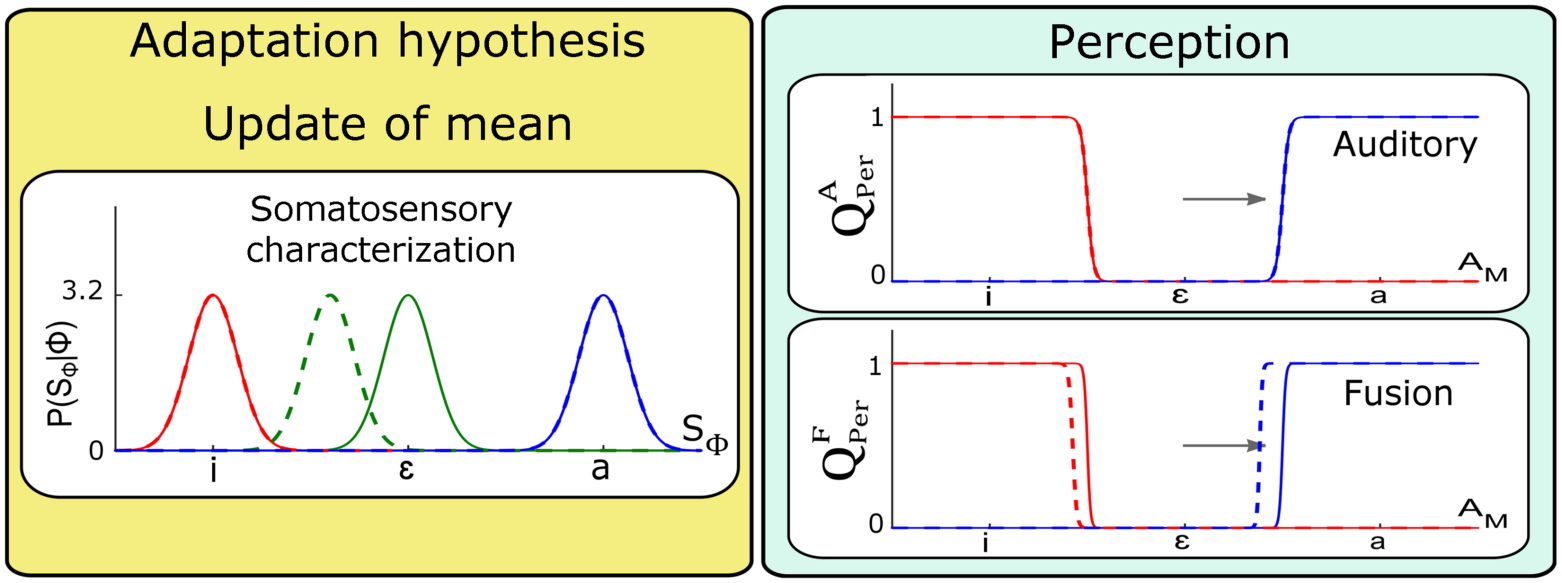
Changes in perception questions resulting from update of the somatosensory characterization of the perturbed vowel *P*(*S*_Φ_ | [Φ = /ɛ/]). The update corresponds to a shift of the mean value $\mu_{S}^{ɛ}$ in the direction of phoneme /i/, as would result from compensation to an auditory perturbation towards /a/ (horizontal arrow). The outcome of simulations before update (solid lines) are superimposed to those after update (dashed lines).

Fig 8 presents the outcome of the two perception questions $Q_{\text{Per}}^{A}$ and $Q_{\text{Per}}^{F}$ after a shift of $\mu_{S}^{ɛ}$ in the direction of /i/. Plots are organized in the same manner as in previous Figures. The left panel illustrates the shift in location of the somatosensory characterization of phoneme /ɛ/ after training, once adaptation is achieved. We observe that the update of *P*(*S*_Φ_ | Φ) does not induce any change in perceptual categories under the auditory pathway hypothesis $Q_{\text{Per}}^{A}$. This is consistent with Eq (11) where the somatosensory characterization *P*(*S*_Φ_ | Φ) is not involved. However, we observe a shift in auditory categorization under the fusion of pathways hypothesis $Q_{\text{Per}}^{F}$. This again is consistent with Eq (13) where the somatosensory characterization term *P*(*S*_Φ_ | Φ) is involved. It must be noted though, that the direction of the perceptual shift is opposite to the perturbation, contrary to the experimental findings reported in S-09 and L-14.

#### Effects of combined update hypotheses

Until now we have only considered individual update hypotheses. The conclusion of these individual evaluations is that the local update of the auditory-motor internal model and the coordinated shift and narrowing of the auditory characterization of phoneme /ɛ/ are the only updates that correctly account for the experimental observations (direction of the shift of the boundaries between perceptual categories and asymmetry of the magnitude of the shift on both sides of vowel /ɛ/) in L-14. These updates are not exclusive and they could be involved simultaneously during adaptation. Hence, in this section we investigate the consequence for the perception questions of the combination of these two updates. Note that we are not discarding an additional update of the somatosensory characterization of phonemes in combination with the two other ones. It could be actually involved and result in a reduction of the perceptual shift induced by any of the two other hypotheses. Since this would only act as an amplification/reduction factor of the main phenomenon, we do not consider it in the remaining of this study.

**Fig 9:**
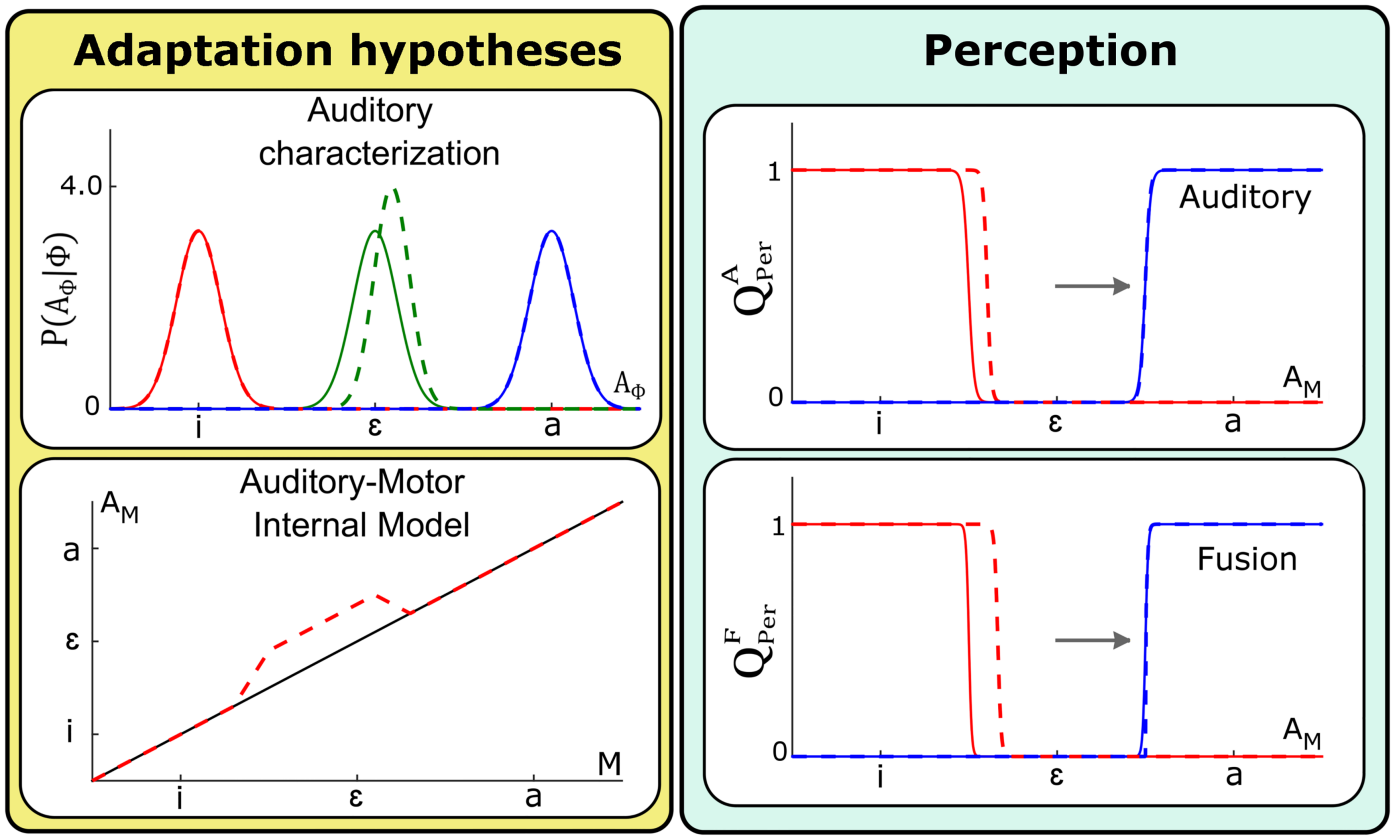
Changes in perception questions resulting from a combined local update of the internal model and an update of the auditory characterization of the perturbed phoneme combining a shift in mean and reduction in variance. Dashed lines correspond to the perturbed condition. The perturbation goes in the direction of /a/ (horizontal arrow).

Fig 9 presents the outcome of the two perception questions $Q_{\text{Per}}^{A}$ and $Q_{\text{Per}}^{F}$ after combination of these update hypotheses. Plots are organized in the same manner as in previous Figures. The two left panels illustrate the changes of the auditory characterization of phoneme /ɛ/ (top) and the auditory-motor mapping (bottom). Consistent with the results presented in Figs 6 and 7, we observe that after these two combined updates both the auditory and the sensory fusion accounts of perception, $Q_{\text{Per}}^{A}$ and $Q_{\text{Per}}^{F}$, result in asymmetric perceptual shifts. These shifts go in the direction of the auditory perturbation and are visible only in the portion of auditory space located with respect to vowel /ɛ/ on the opposite side of the region in which the auditory perturbation was applied, in agreement with the experimental findings reported in L-14.

#### Summary

In summary, based on the results of the simulations presented in this section, we select 3 adaptation hypotheses that, combined with at least one of the two perception hypotheses ($Q_{\text{Per}}^{A}$ or $Q_{\text{Per}}^{F}$), reproduce the key-observations described in L-14 concerning the perceptual boundary shifts after adaptation to the auditory perturbation of vowel /ɛ/:

- $H_{\text{Ad}}^{M}$: the auditory-motor internal model is locally updated during adaptation, in the region where the subject articulates speech during the training phase leading to adaptation. No other update occurs.
- $H_{\text{Ad}}^{\Phi }$: only the auditory characterization of vowel /ɛ/ is modified and this modification involves a combined update of its location and width.
- $H_{\text{Ad}}^{M\Phi }$: both stored knowledge mentioned in $H_{\text{Ad}}^{\Phi }$ and $H_{\text{Ad}}^{M}$ are simultaneously updated.

**Fig 10:**
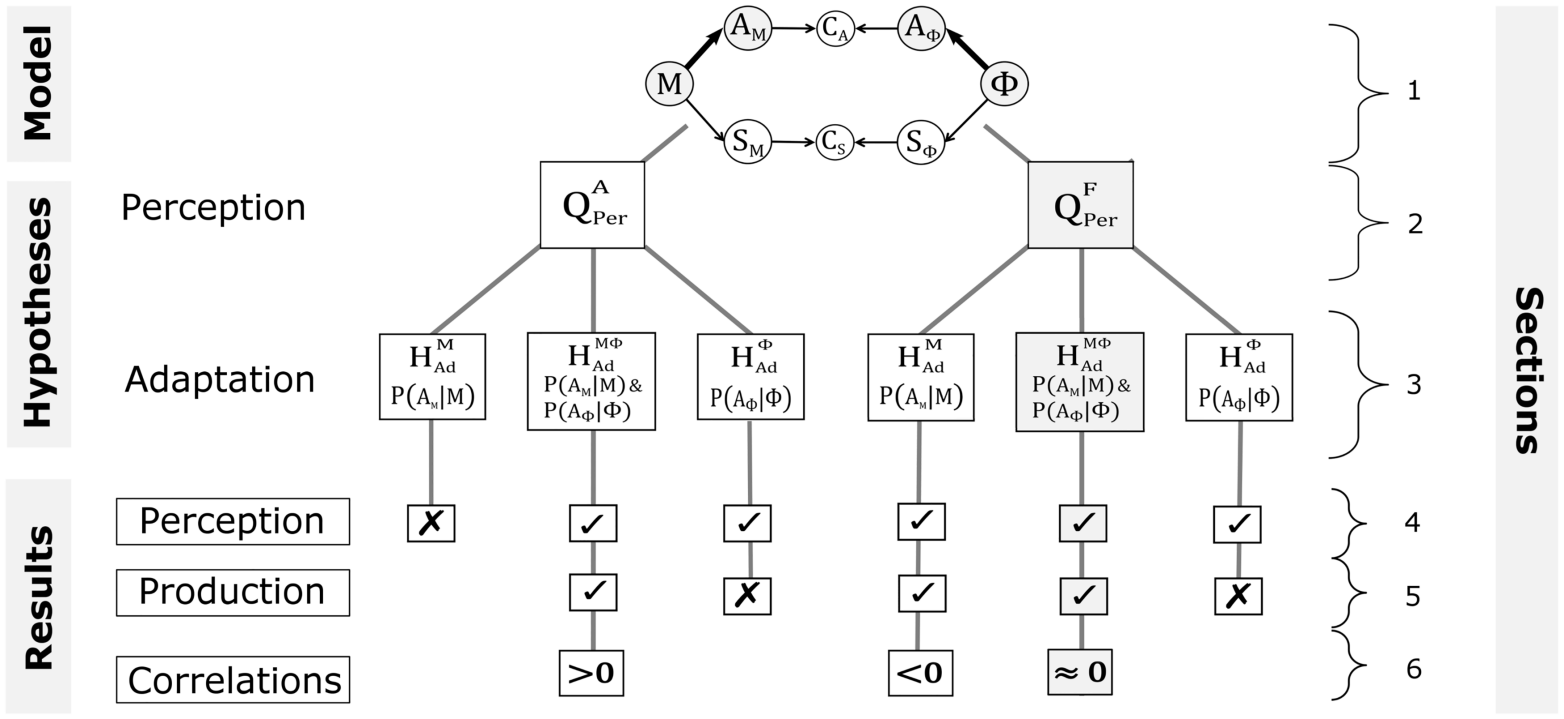
Evaluation of hypotheses about speech perception and adaptation in our modeling work. The model (Section 1 Model definition) enables to define different hypotheses about the sensory pathways involved in speech perception (Section 2 Formulation of speech production and perception questions) and the learning mechanisms involved in adaptation (Section 3 Adaptation hypotheses). Concerning speech perception, we evaluate hypotheses $Q_{\text{Per}}^{A}$ and $Q_{\text{Per}}^{F}$, corresponding to the involvement of the auditory pathway only, or the fusion of somatosensory and auditory pathways. Concerning adaptation, we evaluate hypotheses $H_{\text{Ad}}^{M}$, $H_{\text{Ad}}^{\Phi }$ and $H_{\text{Ad}}^{M\Phi }$, corresponding respectively to an update of the auditor-motor internal model, an update of the auditory characterization of the perturbed phoneme, or both updates simultaneously. Combining hypotheses about perception and adaptation further enables to identify and test different scenarios simulating the experimental paradigm of Lametti et al. [1]. Scenarios leading to perceptual changes incompatible with those observed by Lametti et al. [1] are discarded (x-boxes, Section 4 Evaluation with respect to perception). The remaining scenarios (✓-boxes) are evaluated with respect to their predictions of compensation in production (Section 5 Evaluation with respect to production) and only those that are consistent with results of Lametti et al. [1] are kept. Finally, we evaluate the last scenarios with respect to correlations between perceptual boundary shift and compensation magnitude (Section 6 Evaluation with respect to correlations). The only scenario that matches the no-correlation observation of Lametti et al. [1] is gray-shaded.

Fig 10 illustrates the different stages of our evaluation process. The three selected hypotheses are represented on the third level of Fig 10 from the top. The fourth level represents the outcomes of the evaluation of each hypothesis with respect to perception. The two last levels will be discussed in the next sections.

### Evaluation with respect to production

Let us now evaluate the effect of the three previous adaptation hypotheses, $H_{\text{Ad}}^{M}$, $H_{\text{Ad}}^{\Phi }$ and $H_{\text{Ad}}^{M\Phi }$ with respect to the production question $Q_{\text{Prod}}^{F}$.

#### Evaluation of $H_{\text{Ad}}^{M}$

**Fig 11:**
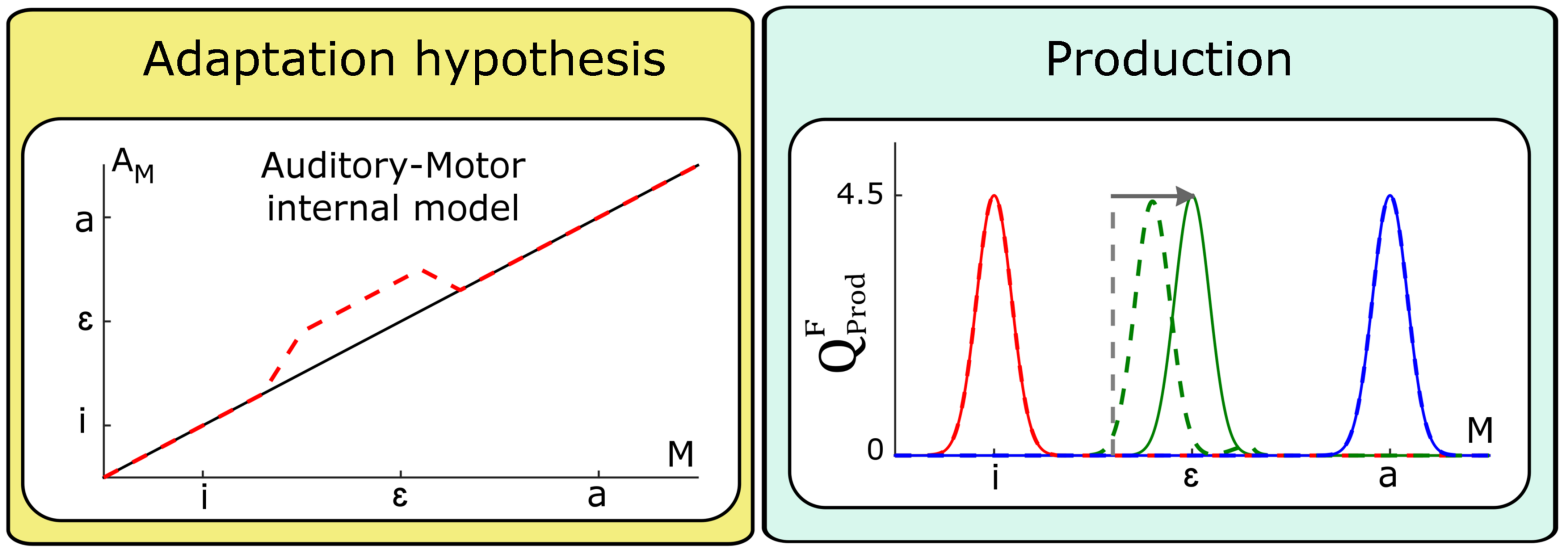
Changes in production question $Q_{\text{Prod}}^{F}$ resulting from a local update of the auditory-motor internal model *P*(*A*_*M*_ | *M*). The update magnitude is equal to the perturbation magnitude (horizontal arrow). The vertical dashed line indicates the shift in control space that would result in complete compensation.

Fig 11 presents the outcome of the planning process $Q_{\text{Prod}}^{F}$ (right panel) before (solid lines) and after (dashed lines) updating the internal model. We observe a shift of the distribution of the motor commands selected for vowel /ɛ/ after adaptation, which is in a direction opposite to the perturbation (shift toward /i/, when the auditory perturbation goes toward /a/), in agreement with the reported compensatory behavior.

Consistent with numerous experimental findings [24, 29, 52–54] our model predicts that the compensation for the perturbation of the auditory feedback is not complete. Yet, in the model, the local update of the internal model has been designed in order to enable a full compensation (the magnitude of the change matches the magnitude of the auditory perturbation). However, full compensation does not occur, because the speech planning process takes in consideration both the auditory and the somatosensory characterization of the phoneme. Full compensation would enable a perfect achievement of the auditory characteristics, but at the price of such a large change of the motor commands, that the corresponding somatosensory consequences would not be compatible any longer with the specified somatosensory characterization of the phoneme. Hence, the incomplete compensation is the result of a compromise between the requirements in terms of auditory and somatosensory characteristics. Note as well that the compensatory change in production is restricted to the vowel /ɛ/. This is a consequence of the locality of the internal model update, which is consistent with experimental observations of transfer of motor learning in speech production [49, 55].

#### Evaluation of $H_{\text{Ad}}^{\Phi }$

**Fig 12:**
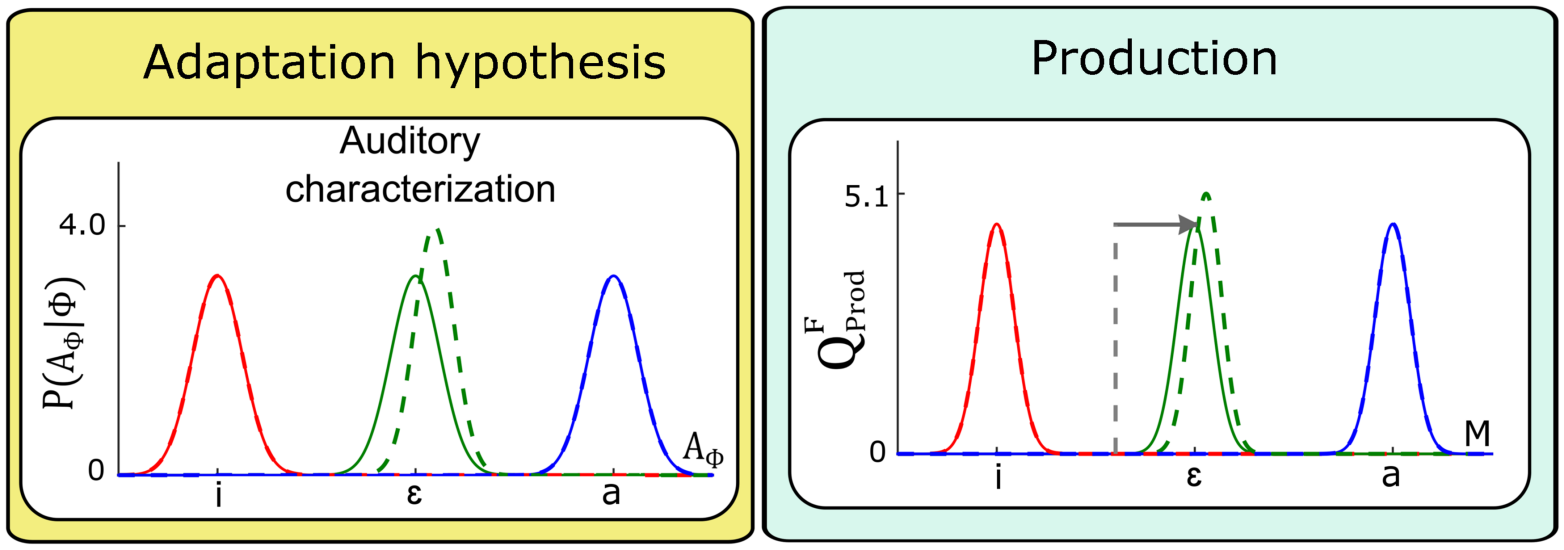
Changes in production question $Q_{\text{Prod}}^{F}$ resulting from an update of the auditory characterization of phoneme /ɛ/ *P*(*A*_Φ_ | [Φ = /ɛ/]). The update correspond to a combined shift of mean, $\mu_{A}^{ɛ}$ and reduction in standard-deviation $\sigma_{A}^{ɛ}$ as previously defined (see Fig 11 for additional details).

Fig 12 presents the outcome of the planning process $Q_{\text{Prod}}^{F}$ (right panel) before (solid lines) and after (dashed lines) updating the auditory characterization of vowel /ɛ/, according to the adaptation hypothesis $H_{\text{Ad}}^{\Phi }$. Since, in the context of this hypothesis, all the other representations are unchanged, in particular the auditory-motor internal model, this change of the auditory characterization of vowel /ɛ/ toward vowel /a/ induces a change of the motor commands that is also toward the articulation of /a/, i.e. in the same direction as the auditory perturbation. This is contrary to the compensatory behavior reported in all the experimental studies involving a perturbation of the auditory feedback.

#### Evaluation of $H_{\text{Ad}}^{M\Phi }$

**Fig 13:**
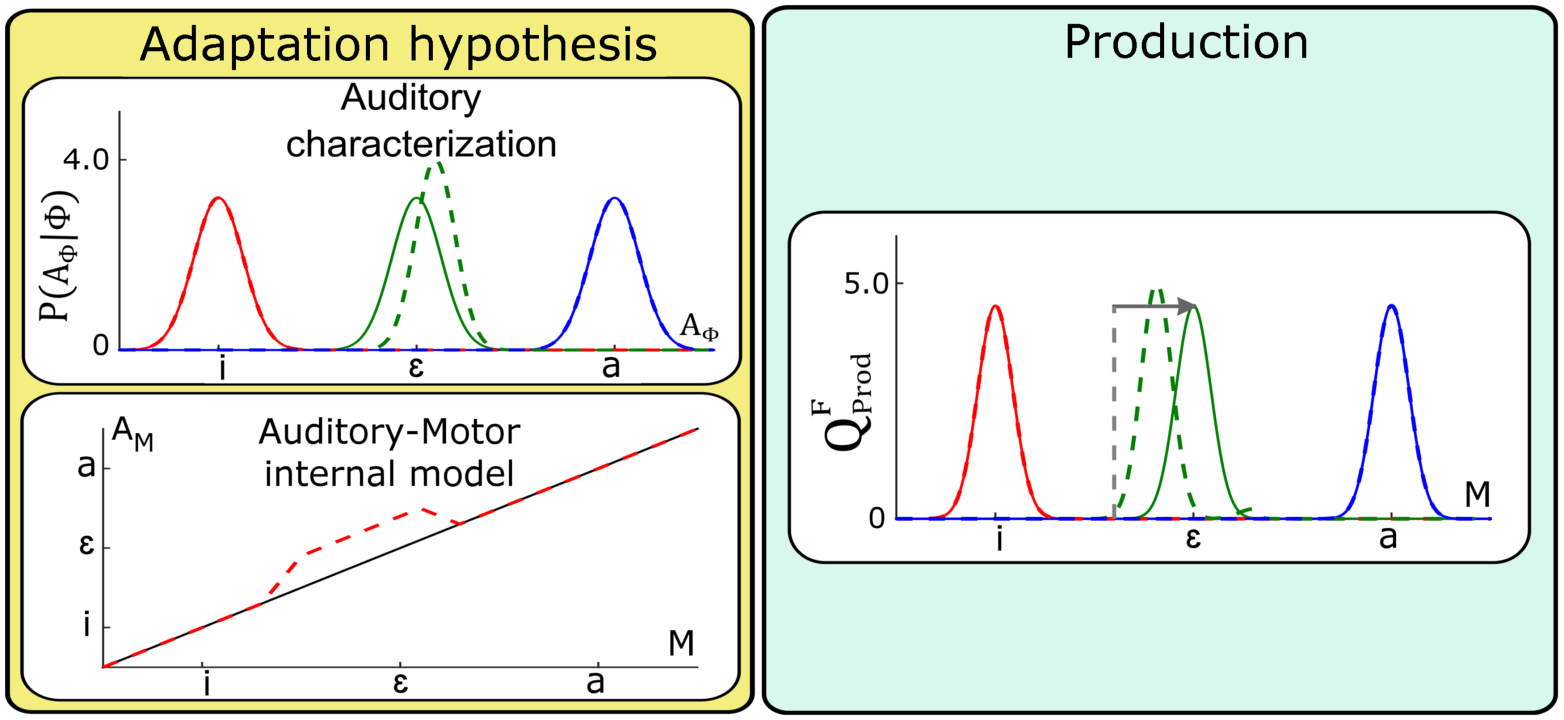
Changes in production question $Q_{\text{Prod}}^{F}$ resulting from the combination of the local update of the internal model and the update of the auditory characterization of vowel /ɛ/. See Fig 11 for additional details.

Fig 13 presents the outcome of the planning process $Q_{\text{Prod}}^{F}$ (right panel) before (solid lines) and after (dashed lines) the combined updates of the auditory-motor internal model and the auditory characterization of vowel /ɛ/, according to the adaptation hypothesis $H_{\text{Ad}}^{M\Phi }$. We observe that, after these two combined updates, the outcome of the planning process (right panel) is shifted in a direction opposite to the perturbation, in agreement with the reported compensatory behavior. Similarly to the previous evaluation of $H_{\text{Ad}}^{M}$, comparing the magnitude of the shift with the amplitude of the perturbation (horizontal arrow) indicates that compensation is not complete. In the present case, the incomplete compensation has a double origin. The shift of the auditory characterization of the perturbed phoneme is an explanation for this phenomenon since this change reduces the need to change articulation in order for the production to match this characterization. In addition, as for $H_{\text{Ad}}^{M}$ in Fig 11, compensation is incomplete due to the fact that the new auditory-motor relation leads to auditory and somatosensory states that cannot simultaneously satisfy the two sensory characterizations of phonemes.

#### Summary

From the three selected adaptation hypotheses, only $H_{\text{Ad}}^{M}$ and $H_{\text{Ad}}^{M\Phi }$ are compatible with the compensatory change in production observed in experimental studies. Altogether, as illustrated in Fig 10, we are hence left with three combined perception–adaptation hypotheses that all reproduce the experimental results in perception and production reported in L-14:

- $Q_{\text{Per}}^{F}⊕H_{\text{Ad}}^{M}$: auditory perception is based on the fusion of auditory and somatosensory pathways ($Q_{\text{Per}}^{F}$) and only the auditory-motor internal model is locally updated during adaptation ($H_{\text{Ad}}^{M}$).
- $Q_{\text{Per}}^{A}⊕H_{\text{Ad}}^{M\Phi }$: auditory perception is based only on the auditory pathway ($Q_{\text{Per}}^{A}$) and both the auditory-motor internal model and the auditory characterization of the perturbed vowel are modified, with a local update for the first and a combined shift and narrowing for the second ($H_{\text{Ad}}^{M\Phi }$).
- $Q_{\text{Per}}^{F}⊕H_{\text{Ad}}^{M\Phi }$: auditory perception is based on the fusion of sensory pathways ($Q_{\text{Per}}^{F}$) and both the auditory-motor internal model and the auditory characterization of the perturbed vowel are modified, with a local update for the first and a combined shift and narrowing for the second ($H_{\text{Ad}}^{M\Phi }$).

**Fig 14:**
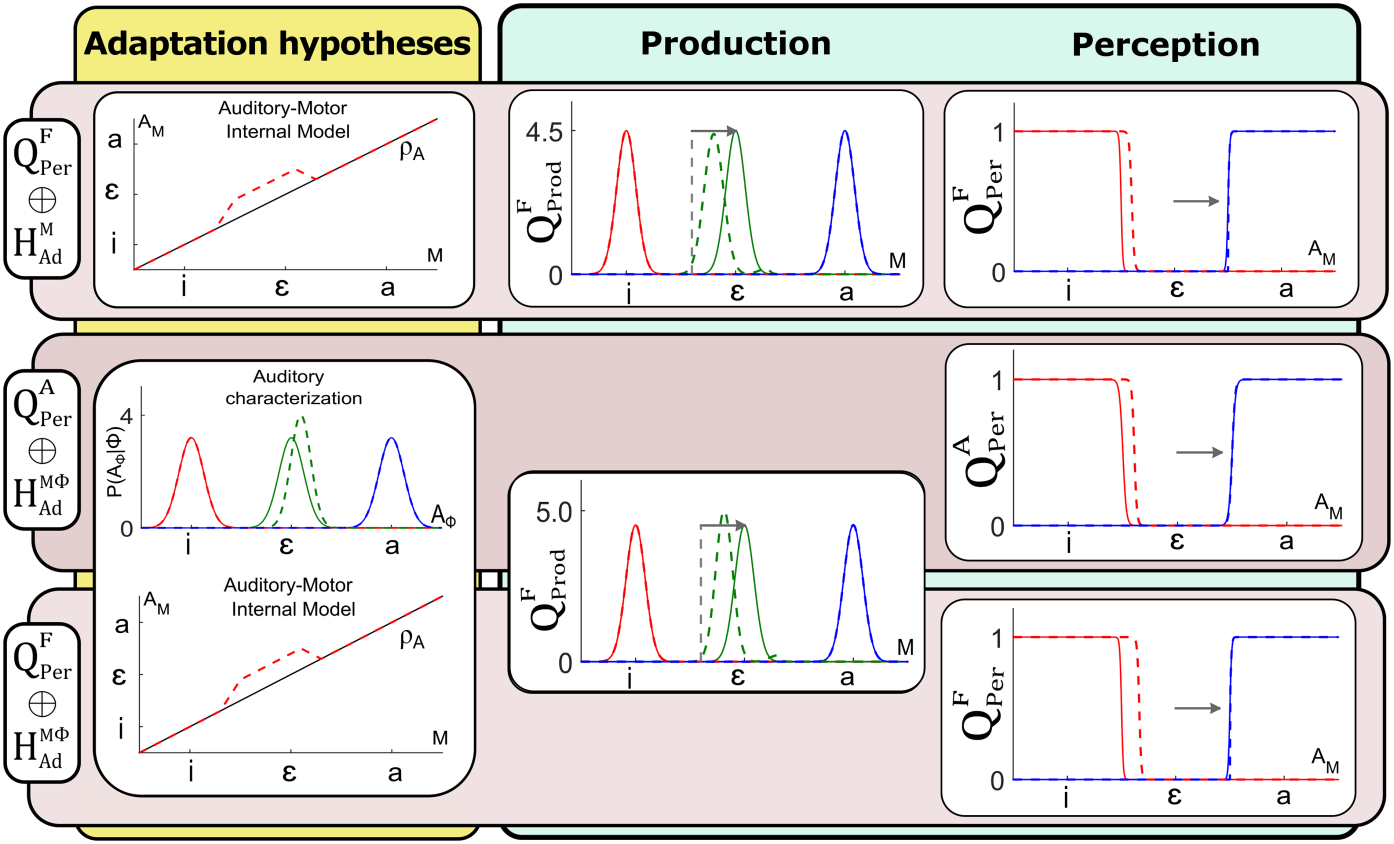
Summary of simulations under each of the three combined hypotheses accounting for the results in L-14.

Fig 14 summarizes the corresponding results in production and perception for each of these three selected hypotheses.

As highlighted above in Section “Update of the auditory-motor internal model *P*(*A*_*M*_ | *M*)”, the asymmetry of the perceptual boundary shift explained by these three hypotheses is sensitive to the particular values of the parameters involved in the updates according to either hypothesis $H_{\text{Ad}}^{M}$ or hypothesis $H_{\text{Ad}}^{M\Phi }$. Other parameter values can lead to boundary changes in both sides of the auditory continuum. The apparent contradiction between studies S-09 and L-14, highlighted in Section “Summary of experimental results we aim at modeling”, could thus be interpreted in this context. Refer to Supporting information S5 Text for variations around this theme.

### Evaluation with respect to correlations

These three combined hypotheses are equivalent in terms of the qualitative effects predicted with respect to changes in production and perception; they all account for incomplete compensation and for the asymmetric perceptual boundary shift in the direction of perturbation. However, the magnitudes of the perceptual boundary shift and of the motor command shift associated with the compensation differ across the three hypotheses. Experimental studies display large differences across subjects in their capacity to compensate for a perturbation of the auditory feedback [56–58]. Moreover, in L-14 and S-09 subjects differ in the amount of perceptual boundary shift induced by adaptation to the perturbation. If, as suggested in L-14, the perceptual change is mainly due to a change in motor functions, one would expect that subjects who compensate more would exhibit a greater perceptual boundary shift. However, no significant correlation between these two phenomena was found in L-14.

In the present section we focus on this question. First, we identify possible origins for the reported differences concerning the amount of compensation and perceptual shift among subjects. Then, we implement these origins under each of the three combined hypotheses and evaluate their predictions in terms of the correlations between compensation magnitudes and amount of perceptual boundary shift.

#### Hypotheses on the origins of variability in the magnitude of compensation and perceptual shift

Up to now, we have compared simulations in which for each hypothesis we have arbitrarily chosen a unique set of new parameters for the piece of knowledge that is assumed to be modified during the adaptation process. However, since compensation and adaptation mechanisms in presence of perturbation are highly subject-dependent, we can see our approach as the modeling of a specific subject behavior. In this section we will consider some variations in the changes associated to adaptation in order to investigate the possible consequences of inter-subject variability in the compensation/adaptation process on the categorical boundary shifts in perception.

The adaptation assumptions selected in Section “Effects of combined update hypotheses” involved the local update of the auditory-motor internal model *P*(*A*_*M*_ | *M*) and the update of the auditory characterization of the perturbed phoneme *P*(*A*_Φ_ | [Φ = /ɛ/]). Therefore, inter-subject differences in adaptation can be attributed in the model to different update magnitudes in either of these two terms. These different magnitudes may result from inter-subject differences in learning rates, in novelty or error detection, etc. This leads to the two following hypotheses:

- $H_{\text{Var}}^{M}$: subjects differ in the magnitude of update of their auditory-motor internal model, *P*(*A*_*M*_ | *M*), some of them achieving a complete update and some others only a partial update.
- $H_{\text{Var}}^{\Phi }$: subjects differ in the amount of shift of their auditory characterization of the perturbed phoneme, *P*(*A*_Φ_ | [Φ = /ɛ/]) (still assuming the relation between mean and variance used in Section “Update of the auditory characterization *P*(*A*_Φ_ | Φ)”, i.e such that the perceptual boundary shift is present only on one side of the auditory continuum).

In addition to the two previous hypotheses, we previously noted that, in our model, incomplete compensation resulted from a trade-off between the constraints associated with the auditory and the somatosensory characterizations of the phonemes, which are no longer compatible after adaptation. It is important to point out that the result of this trade-off depends only on the relative strength of the constraint imposed by each sensory pathway. In our previous simulations, both sensory constraints were equivalent (same values of parameters characterizing the Gaussian distributions and linear relation between the two sensory domains), meaning that perturbations to each modality would be equally compensated. However, individual differences in the amount of compensation to auditory and somatosensory perturbations have been reported in speech production: subjects that adapt more to one sensory perturbation tend to adapt less to the other [59]. This has been suggested as evidence that some subjects may rely more on the auditory modality and others more on the somatosensory modality. Such sensory preferences could originate from individual differences in the sensitivity to each kind of sensory feedback [60], which can be modeled in line with the suggestions of Perkell et al. [52, 61], by differences in the parameters $\sigma_{A}^{\phi }$ and $\sigma_{S}^{\phi }$. Small $\sigma_{A}^{\phi }$ (resp. $\sigma_{S}^{\phi }$) values means that the auditory (resp. somatosensory) characterization of the phoneme is very accurate and that the subject strongly relies on this sensory pathway. Large $\sigma_{A}^{\phi }$ (resp. $\sigma_{S}^{\phi }$) values means either that the sensory characterization is quite inaccurate or that the subject does not rely much on this sensory pathway.

Therefore, we consider a third possible hypothesis concerning the origin of the reported differences in compensation between subjects:

- $H_{\text{Var}}^{\sigma }$: subjects differ in the relative precision of their sensory characterizations of phonemes. Some may have greater values of parameter $\sigma_{A}^{\phi }$ compared to $\sigma_{S}^{\phi }$ and *vice versa*.

#### Implementing hypotheses and exploring correlations between the magnitude of compensation and perceptual shift

The three previous hypotheses represent three possible origins of the reported differences in the way subjects adapt to perturbations. These hypotheses are not exclusive and all of them may be involved simultaneously. However, in order to simplify the presentation of the results, we firstly focus on the combined effects of hypotheses $H_{\text{Var}}^{M}$ and $H_{\text{Var}}^{\Phi }$. In other words, we first implement simulations combining different values of update of the auditory-motor internal model ($H_{\text{Var}}^{M}$), and different values of shift of the auditory characterization of the perturbed phoneme ($H_{\text{Var}}^{\Phi }$). Hence, in this first set of simulation we ignore hypothesis $H_{\text{Var}}^{\sigma }$ and keep values of $\sigma_{A}^{\phi }$ and $\sigma_{S}^{\phi }$ equal.

**Fig 15:**
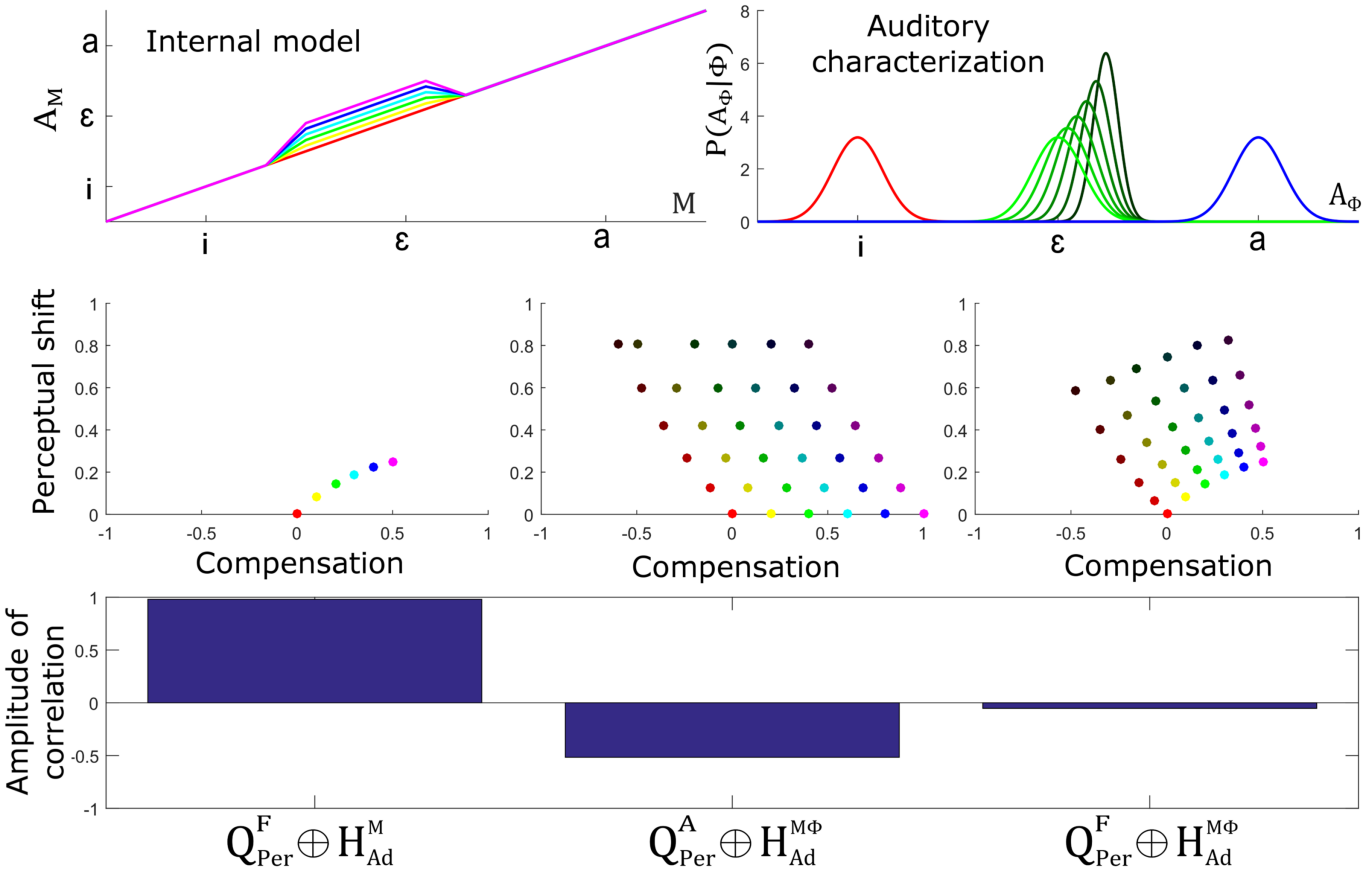
Relation between amplitude of perceptual boundary shift and amount of compensation for hypothesis $H_{\text{Var}}^{M}$ and hypothesis $H_{\text{Var}}^{\Phi }$. Top panels: considered amplitude of update in the auditory-motor internal model (left) and the amplitude of shift in the auditory characterization of the perturbed phoneme (right). Middle panels: relation between degree of compensation and amount of perceptual shift for each adaptation hypothesis in the case of a local update of the internal model. Colors correspond to the different magnitudes of internal model updates, as indicated in the top left panel. Darkness indicates the amplitude of shift of the auditory characterization of the perturbed phoneme, as indicated in the top right panel. Bottom panel: corresponding amplitude of correlations.

Fig 15 presents the outcome of simulations for different updates of the auditory-motor internal model and different shifts of the auditory characterization of the perturbed phoneme, for a magnitude of the perturbation representing 40% of the distance between neighboring phonemes and towards phoneme /a/. For the internal model, we specified six update amplitudes in order to enable a compensation varying gradually from 0% to 100% of the magnitude of perturbation (when no influence of other factors reduces compensation) (top left panel of Fig 15). For the auditory characterization of vowel /ɛ/, we implemented six values of parameter $\sigma_{A}^{ɛ}$, evenly distributed from the original value, used in the normal condition, to half of this value. The six corresponding values for $\mu_{A}^{ɛ}$ (top right panel of Fig 15) were computed from the relation that was already used in Section “Update of the auditory characterization *P*(*A*_Φ_ | Φ)” (as stated in hypothesis $H_{\text{Var}}^{\Phi }$).

Middle panels present the magnitude of compensation and perceptual shifts resulting from the combination of previous updates under the three combined hypotheses, $Q_{\text{Per}}^{F}⊕H_{\text{Ad}}^{M}$ (left panel of Fig 15), $Q_{\text{Per}}^{A}⊕H_{\text{Ad}}^{M\Phi }$ (middle panel of Fig 15) and $Q_{\text{Per}}^{F}⊕H_{\text{Ad}}^{M\Phi }$ (right panel of Fig 15). Colors correspond to the different magnitudes of internal model update and darkness indicates the amplitude of shift of the auditory characterization of the perturbed phoneme, as indicated by plots in the top panels. X-axis represents the magnitude of compensation in units of the perturbation but in the opposite direction. In other words, value 1 corresponds to a shift in production of the same magnitude but opposite direction of the perturbation (complete compensation), value 0 corresponds to no compensation and value -1 corresponds to a shift in production of the same magnitude and same direction as the perturbation. Y-axis represents the amount of perceptual shift in units of the perturbation and in the same direction.

The bottom panel indicates the correlation coefficient between compensation and perceptual shift for the set of data points obtained from the different simulations under each of the three combined hypotheses. Simulations assuming hypothesis $Q_{\text{Per}}^{F}⊕H_{\text{Ad}}^{M}$ (left middle panel in Fig 15) show a noticeable positive correlation between magnitude of compensation and perceptual shift. In order to understand this result, it is important to remember that in the context of hypothesis $Q_{\text{Per}}^{F}⊕H_{\text{Ad}}^{M}$ only an update of the auditory-motor internal model is assumed. Hence, hypothesis $H_{\text{Var}}^{\Phi }$ does not apply, and only the effect of inter-subject differences in internal model updates (hypothesis $H_{\text{Var}}^{M}$) can be considered. The magnitude of the articulatory changes associated with compensation is strongly related with the magnitude of the changes provided to the internal model (displacement along the horizontal axis in the figure). Our simulations show that the perceptual boundary shift increases with the magnitude of the changes, but non monotonously: it increases first and becomes stable after. This “saturation” effect is due to the fact that the update of the internal model is local. Altogether, the influence of the update of the internal model in production and perception results in a noticeable positive correlation between the amount of compensation and perceptual shift, contrary to what was reported in L-14.

Simulations assuming hypothesis $Q_{\text{Per}}^{A}⊕H_{\text{Ad}}^{M\Phi }$ for the perceptual boundary shift (center panel in Fig 15) show a negative and moderate correlation between amount of compensation and perceptual shift. It should be reminded that in the context of hypothesis $Q_{\text{Per}}^{A}⊕H_{\text{Ad}}^{M\Phi }$ adaptation induces both a local update of the motor-auditory internal model and an update of the auditory characterization of vowel /ɛ/, and that perception only involves the auditory pathway. In the absence of any other constraint, the magnitude of the update of the auditory-motor internal model (hypothesis $H_{\text{Var}}^{M}$) strongly determines the magnitude of the compensation. We have shown above that, when perception only involves the auditory pathway, the update of the auditory-motor internal model has no influence of the perceptual boundary shift. Hence, the update of the internal model does not induce any correlation between the amount of compensation and the magnitude of the perceptual boundary shift. This can be seen in the present simulations where data points corresponding to a given location of the auditory characterization (same darkness) but different values of update of the internal model (different colors) are aligned horizontally.

On the contrary, a shift in the auditory characterization of vowel /ɛ/ has a direct impact on the perceptual boundary shift (positive correlation) and on the amount of compensation (the larger the shift, the smaller the amount of compensation). Thus inter-subject differences in the magnitude of the shift of the auditory characterization of vowel /ɛ/ (hypothesis $H_{\text{Var}}^{\Phi }$) result in a negative correlation between the amount of compensation and the magnitude of the perceptual boundary shift. Altogether, in the context of hypothesis $Q_{\text{Per}}^{A}⊕H_{\text{Ad}}^{M\Phi }$, the combination of hypotheses $H_{\text{Var}}^{M}$ and $H_{\text{Var}}^{\Phi }$ results in a mild negative correlation between the amount of compensation and the magnitude of the perceptual boundary shift, contrary to what was reported in L-14.

Simulations assuming hypothesis $Q_{\text{Per}}^{F}⊕H_{\text{Ad}}^{M\Phi }$ for the perceptual boundary shift (right middle panel in Fig 15) show an almost vanishing correlation between amount of compensation and perceptual boundary shift. Hypothesis $Q_{\text{Per}}^{F}⊕H_{\text{Ad}}^{M\Phi }$ can be roughly seen as combining $Q_{\text{Per}}^{F}⊕H_{\text{Ad}}^{M}$ and $Q_{\text{Per}}^{A}⊕H_{\text{Ad}}^{M\Phi }$. Since $Q_{\text{Per}}^{F}⊕H_{\text{Ad}}^{M}$ induces a positive correlation and $Q_{\text{Per}}^{A}⊕H_{\text{Ad}}^{M\Phi }$ a negative one, the combination of these two influences in $Q_{\text{Per}}^{F}⊕H_{\text{Ad}}^{M\Phi }$ tends to counterbalance each other, resulting in a much smaller correlation than the two previous ones. For a given shift of the auditory characterization of vowel /ɛ/ (same darkness) simulations with different updates of the internal model (different colors) result in a similar pattern as in the simulations assuming $Q_{\text{Per}}^{F}⊕H_{\text{Ad}}^{M}$.

However two variations of this pattern can be observed when the shift of the auditory characterization increases. First, the non-linearity induced by the locality of update of the internal model (see above) disappears when the shift increases (darkest *versus* lighter data points). This is due to the fact that the shift in the auditory characterization brings the boundary between phonemes closer to the center of the updated region and reduces the influence of the limits of the local update. (Simulations assuming the general update of the internal model were performed in order to clarify which part of the effects arises from our locality assumption. The obtained results show the same key-properties for both updates of the internal model, which indicates that the obtained pattern of correlations is not an artifact of the particular choice of our local update assumption.)

The second difference is that the slope of the relation between perceptual shift and compensation reduces for greater shifts of the auditory characterization. This is due to the fact that the increase in the magnitude of the shift goes together with a decrease in the width of the auditory characterization of vowel /ɛ/. This results in a stronger influence of the auditory pathway relative to the somatosensory one. Since the influence of the internal model on the perceptual boundary shift is mediated through the somatosensory pathway, the magnitude of the effect reduces when the auditory pathway is stronger. This is consistent with the horizontal alignment obtained under $Q_{\text{Per}}^{A}⊕H_{\text{Ad}}^{M\Phi }$ where the somatosensory pathway is assumed not to contribute to perception.

In summary, hypothesis $Q_{\text{Per}}^{F}⊕H_{\text{Ad}}^{M\Phi }$ is more in line with the lack of correlation between compensation and perceptual shift reported in L-14.

**Fig 16:**
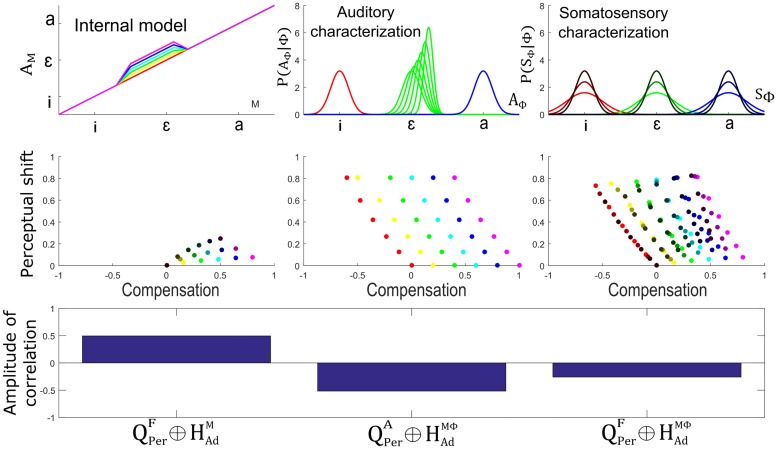
Influence of the combination of hypotheses $H_{\text{Var}}^{M}$, $H_{\text{Var}}^{\Phi }$ and $H_{\text{Var}}^{\sigma }$ on the relation between compensation and perceptual boundary shift. Changes implemented for the internal model and the auditory characterization are the same as in Fig 15. They are illustrated in the top left and top middle panels. In addition to the previous simulations where parameters of the sensory characterizations, $\sigma_{S}^{\phi }$ and $\sigma_{A}^{\phi }$, where both equal, here we implement two additional values of parameters $\sigma_{S}^{\phi }$, corresponding to greater variance of the somatosensory characterization, and therefore reduced weight of the somatosensory pathway. The three values of $\sigma_{S}^{\phi }$ implemented are $\frac{1}{8}$ (equal weights of sensory pathways), $\frac{1}{6}$ and $\frac{1}{4}$ (smaller weight for the somatosensory pathway) of the distance between neighboring phonemes. The corresponding changes of the somatosensory characterization are illustrated in the top right panel. Darkness of the colors indicates an increase of the weight of the auditory pathway relatively to the somatosensory pathway.

We now consider the additional influence of hypothesis $H_{\text{Var}}^{\sigma }$, assuming variable relative precision of the sensory characterizations of phonemes across subjects. We implemented the same simulations as above for different values of parameter $\sigma_{S}^{\phi }$, as illustrated in the top right panel of Fig 16. We retained values of $\sigma_{S}^{\phi }$ only greater or equal to the value of $\sigma_{A}^{\phi }$ (corresponding to preference on the auditory pathway as compared to the somatosensory pathway), in order to be consistent with the fact that in L-14 only subjects who showed significant compensation to auditory perturbations were kept. Notice that simulations implementing reciprocal values for $\sigma_{A}^{\phi }$ and $\sigma_{S}^{\phi }$ were also performed. Results are qualitatively similar to those presented below, indicating that they are not a consequence of the particular asymmetric choice implemented here. In Fig 16, the level of darkness of the colors indicates an increasing precision of the somatosensory regions.

Results are consistent with the idea that relative precision of sensory characterizations modulates the influence of each sensory pathway: wider somatosensory characterizations (light colors) are associated with a larger influence of the auditory pathway. As a consequence, in the case of $Q_{\text{Per}}^{F}⊕H_{\text{Ad}}^{M}$ (Fig 16, left column) this results in smaller slopes in the relation between perceptual shift and compensation (middle horizontal panel), and decreases the positive correlation between compensation and perceptual shift accordingly (lower horizontal panel). However, for hypothesis $Q_{\text{Per}}^{A}⊕H_{\text{Ad}}^{M\Phi }$ (Fig 16, center column) in which the somatosensory pathway is assumed not to contribute to perception, varying somatosensory weight has no consequence on perceptual shift (middle horizontal panel) nor on the resulting correlation (lower horizontal panel). Finally, we observe that hypothesis $H_{\text{Var}}^{\sigma }$ has a small impact on the correlation coefficient obtained assuming $Q_{\text{Per}}^{F}⊕H_{\text{Ad}}^{M\Phi }$ (Fig 16, right column) which remains close to zero.

In summary, including hypothesis $H_{\text{Var}}^{\sigma }$ in our simulations, which corresponds to the implementation of individual differences on the weighting of each sensory pathway, confirms that the absence of correlation reported by L-14 may be best attributed to hypothesis $Q_{\text{Per}}^{F}⊕H_{\text{Ad}}^{M\Phi }$.

## Discussion

Using our model, implemented in the Bayesian programming framework, we have been able to implement and test different hypotheses concerning speech motor adaptation to perturbed auditory feedback. In this framework, processes are not directly modeled but are derived from a common set of knowledge, which is represented by means of a joint probability distribution. Hence, in this approach, perception and production processes become naturally related since changes to the underlying knowledge may impact them together. Note that this framework is not restricted to speech, but may be of interest in other areas where production and perception processes have been shown to interact (for instance in the arm motor control literature, see Haith et al. [62] and Ito et al. [63] for alternative approaches, see also Gilet et al. [36] in the context of joint modeling of perception and production of isolated cursive letters).

We have applied this framework to study the perceptual changes that result from motor learning in adaptation to an auditory perturbation in speech. To do so, we have proposed a number of hypotheses about the changes to the common underlying knowledge that may result from motor learning and we have investigated how these changes may give rise to the observed changes in perception and production. This approach has allowed us to identify different possible origins that all may contribute to these changes, supporting but also specifying the interpretation proposed by Lametti et al. [1].

Our experimental simulations provide a number of major results: (1) the induced perceptual shift may actually be compatible with either an auditory or a combined auditory and somatosensory characterization of perceptual targets; (2) the incomplete motor response to auditory perturbations may be due to a mixture of components, related to the combined specification of the phonemic targets for speech production in auditory and somatosensory terms; (3) the asymmetry in perceptual compensation observed in L-14 is also compatible with both theoretical frameworks in speech perception, but actually appears to be sensitive to fine tuning of the experimental parameters in the simulations; (4) patterns of correlations between perceptual and motor responses may be driven by various factors that shed a crucial light on final interpretations of the experimental data.

Of course, these simulations quantitatively depend on a number of modeling choices introduced in Section “Selected aspects for modeling”, that are aimed at making simulations tractable and easy to analyze and interpret. This basically includes: (1) the assumption that sensory and motor spaces are one-dimensional, (2) the assumption that sensory-motor mappings are linear (Eqs (2–5, 15 and 16), and (3) the specific tuning of parameters considered in the update hypotheses for adaptation. Still, it is important to stress that the four major results summarized previously have an intrinsic validity, which makes them largely independent of the specific modeling choices. This is due to two major reasons. Firstly, the modeling framework introduced in this work has actually been developed over the years completely independently of the experimental data discussed here. This framework is essentially conceived as a general architecture for formalizing classical assumptions about perceptuo-motor relationships in speech communication [12–14].

Secondly, the four major results appear as general, and likely to be obtained whatever the specific choices in the model. Indeed, the first, second and fourth of these results express direct consequences of the model architecture, in which multisensory fusion (between auditory and somatosensory representations) in speech production and possibly in speech perception naturally result in trading relationships leading to (1) perceptual adaptation in response to the motor adaptation (2) incomplete response to perturbation and (3) various types of correlation patterns between motor and perceptual adaptation. The case of the third result (asymmetry in perceptual compensation) is quite interesting in this respect. Indeed, it is, contrary to the others, largely ad hoc and related to the specific modeling choices (i.e., the precise relation between mean and variance and parameters of the local update of the internal model, see Supporting information S3 and S4 Text). This makes it fragile and probably not very robust experimentally. But this fragility can also be construed as a prediction: it means that asymmetries should vary from one study to the other, and that this observation is probably not as reliable as what was expected by the authors of L-14 (see for instance a recent study by Schuerman et al. [64] where no significant boundary shift was obtained).

Interestingly, the symmetric vs. asymmetric nature of the perceptuo-motor adaptation process should also largely depend on the nature of the motor-to-sensory internal model, and it is quite well-known that the motor-to-sensory relationship is indeed highly nonlinear, and likely to vary greatly depending on the involved region of the motor or sensory space. This could well explain the difference between the study by Lametti et al. [1] on vowels, that shows a lack of perceptual shift in the region of the auditory space related to what subjects heard in presence of the perturbation, and the study by Shiller et al. [2] in which a perceptual shift in the corresponding regions with fricatives was observed.

Finally, with respect to the one-dimensional assumption, including additional dimensions in sensory and motor spaces may certainly bring interesting behaviors, such as trading relations between dimensions in compensation. However, the /i ɛ a/ continuum considered in L-14 can be basically seen as one-dimensional both in the articulatory space in which the location of the highest point of the tongue is controlled along the high/front—low/back dimension thanks to strong correlations between jaw opening and tongue position [65], —and in the acoustic space with correlated variations between F1 and F2 respectively increasing and decreasing from /i/ to /a/ [66]. Therefore, such additional effects would likely bring only a modulatory change to the magnitude of the resulting shifts in production and perception, without changing the general patterns of results in our simulation.

Therefore, we consider that the simulation results presented here have intrinsic validity. As a consequence, it is of interest to discuss them as some new evidence that can be confronted to important questions related to perceptuo-motor adaptation as discussed in the literature. This is what we will do now, around two points that are the nature of perceptual representations and the origins of incomplete compensation, before introducing some predictions and proposals for new experiments in the field.

### Revisiting the interpretation presented in L-14

The first stage of our simulations (Section “Evaluation with respect to perception”) both supports and challenges the interpretation by Lametti et al. [1], whereby their data would provide evidence for the role of motor knowledge in speech perception. On the one hand, hypothesis $Q_{\text{Per}}^{F}⊕H_{\text{Ad}}^{M}$, involving only an update of motor functions, is compatible with their interpretation and in fact also specifies it. Indeed, under this hypothesis a local compensation for the perturbation is required to generate a pattern of perceptual adaptation fitting the asymmetry reported in L-14. On the other hand, in the context of hypothesis $Q_{\text{Per}}^{A}⊕H_{\text{Ad}}^{M\Phi }$, involving both a local update of the auditory-motor internal model and a modification of the auditory characterization of the perturbed phoneme, a pure auditory theory of speech perception ($Q_{\text{Per}}^{A}$) also provides a pattern of perceptual shifts compatible with their data, even including asymmetries that were considered as key in their reasoning against auditory theories. In this case, changes in the auditory characterization of a phoneme, involving a coordinated shift of the center of its characterization and a reduction of its variance, are required to explain their results.

It is important to note that it is not unrealistic to assume that motor learning can induce such coordinated changes. Indeed, the shift in location may be explained by a mechanism aligning the auditory characterization of a vowel with its actual realization in presence of the auditory feedback perturbation. The reduction of variance could be attributed to the well-known selective adaptation phenomenon, as suggested by Kleinschmidt et al. [67]: the repeated exposure to the same sound tends to make listeners more sensitive to variations of this sound. Note that, in S-09, selective adaptation was mentioned in order to explain the small perceptual boundary shift observed in their control group after the repeated exposure to the unaltered fricative /s/.

Therefore, at this stage, both an audio-motor and a pure auditory theory may be compatible with the data in L-14. However, the analysis, based on correlations between the amplitude of the perceptual shift and the magnitude of the compensation, indicates that none of the two previous interpretations is compatible with the observations described in L-14. Only hypothesis $Q_{\text{Per}}^{F}⊕H_{\text{Ad}}^{M\Phi }$, assuming the fusion of sensory pathways in speech perception and adaptation involving the combined updates of the auditory-motor internal model and the auditory characterization of the perturbed phoneme, was compatible with the absence of significant correlation reported in L-14.

In summary, our results support and clarify the initial interpretation of Lametti et al. [1]. By exploiting perceptuo-motor correlations, our results support the claim that both sensory and motor processes intervene in the observed perceptual shift. This result certainly speaks in favor of perceptuo-motor theories of speech perception, though further work should be done in order to better assess the relative contributions of each of these two sets of processes [14].

### Three suggested origins for incomplete compensation

Interestingly, in our model, all possible explanations of the link between motor learning and perceptual boundary shift are associated with incomplete compensation for the perturbation, even if the magnitude of the local update of the auditory-motor internal model fully matches the amplitude of the auditory perturbation. This is an important prediction of our model, since incomplete compensations have been systematically observed in all experiments involving a perturbation of the auditory feedback during speech production.

Three mechanisms can indeed be at the origin of incomplete compensation. Firstly, if motor learning induces only an update of the auditory-motor internal model in the context of a bi-modal speech production process, incomplete compensation comes from the interaction between the somatosensory and the auditory specifications of vowels. Secondly, if motor learning also induces a shift and a reduction of variance of the auditory specification of the perturbed phoneme, this provides an additional counter-influence to compensation and the magnitude of the change of the auditory characterization contributes to incomplete compensation. Thirdly, in all cases, if motor learning induces an update of the auditory-motor internal model, the magnitude of this update influences the extent of the compensation: the smaller the update, the more incomplete the compensation.

All these potential explanations of incomplete compensation for perturbations of the auditory feedback have been previously suggested in the literature. In particular, Katseff et al. [68], among other hypotheses, compared the respective influences on the compensation magnitude of a possible interaction between the auditory and the somatosensory feedback *versus* of a possible shift of the auditory region characterizing the pronounced phoneme. They concluded that behavioral data about compensation for auditory perturbation published in the literature (including those in S-09) are more compatible with an interaction between the two sensory feedbacks.

According to them, in the case of the data in S-09, if the perceptual boundary shift is due to a shift of the auditory characterization of the perturbed phoneme, this latter shift should have the same small amplitude as the former one. Such a small shift of the auditory characterization of the phoneme could not explain the large magnitude of the reduction in compensation.

Our results allow us to qualify their conclusion. Indeed, we have shown that when the shift of the auditory characterization is associated with a reduction of its variance, the magnitude of this shift can be much larger than the magnitude of the perceptual boundary shift. In this case, the shift of the auditory characterization of the perturbed phoneme would perfectly account for the amplitude of the compensation.

### Caveats and future directions

At this stage, we have at our disposal a modeling framework to account for the links between production and perception processes. However, the present work focuses on adaptation, by comparing states before and after learning. Investigating the dynamic process occurring during adaptation could provide interesting further insights into the phenomena associated with adaptation. More specifically, the manner with which compensation strategies integrate sensory feedback would inform about the way the sensory-motor characteristics of speech production are updated during the learning phase. For instance, the completeness of compensation appears to be dependent on the amplitude of the perturbation: greater amplitudes of perturbation induce greater sensory errors which appear to result in smaller percentage of total compensation compared to smaller sensory errors. This result seems to be a general property of sensorimotor learning: indeed it has been reported for speech [53, 68], for eye and arm movements [69, 70] and even for bird song [71]. Still, the mechanisms responsible for this decrease in relative adaptation in the case of increasing sensory errors remain unclear.

Our model, in its current state, does not address this question, since it deals only with the consequences of parameters updates, and not with how these updates happen during the learning phase. However, the three possible origins of incomplete compensation (discussed in Section “Three suggested origins for incomplete compensation”) actually suggest three possible mechanisms whereby different magnitudes of sensory error would result in different degrees of compensation completeness. First, at the level of the sensory motor mappings, larger sensory errors may drive slower update in order to avoid a faulty reorganization of the learned mapping in the case of totally unexpected and inappropriate sensory signals (see for instance the work of [72] for a modeling approach in line with this idea). Second, at the level of the relative weighting of sensory pathways, the magnitude of sensory errors could disadvantage the pathway with larger errors, assuming that large unexpected errors would arise from inaccurate sensors, which would then be considered unreliable. Finally, at the level of the sensory characterization of the target, larger sensory perturbations may drive larger shifts of the intended target, resulting in smaller amounts of compensation compared to baseline. Each of these hypotheses deserves more careful analysis in light of the existing experimental data: for example, the third hypothesis appears unlikely, since, after the removal of the perturbation, subjects usually return close to the original baseline. Still, these three hypotheses definitely deserve further experimental focus.

Interestingly, our model gives different predictions for these three hypotheses. For instance, if larger sensory errors disadvantage the weighting of one of the sensory pathways, the model would predict that subjects would begin to compensate more for perturbations in the other sensory modality. Such sensory preferences have been reported previously in speech production [59]; however, to our knowledge, no study has explored the possibility that these preferences may be experimentally modulated by providing larger perturbations to one of the sensory modalities. On the other hand, if sensory errors only influence the update of the sensory-motor mapping or the shift of the sensory characterization of the target, the model would predict no influence of the amount of compensation to perturbations on the other sensory modality. Furthermore, evaluating the influence of the amplitude of perturbation with respect to the resulting perceptual shift could also allow distinguishing between these last two hypotheses. Indeed, if larger sensory errors decrease the update of the sensory-motor mapping, the model would predict a decrease in the amount of perceptual shift, whereas the contrary would happen if larger sensory errors drive greater shifts in the sensory characterization of the target.

Furthermore, as we suggested above, the present model is not limited to the study of auditory perturbations, and investigating the consequences of somatosensory perturbations would allow further evaluation of its pertinence. Indeed, another interesting prediction of the model is that, if adaptation to a somatosensory perturbation updates the somatosensory-motor mapping, it would also induce a boundary shift in the auditory categorization of the perturbed phoneme (but in an opposite direction to perturbation, contrary to the case of auditory perturbations). Such perceptual change following adaptation to a somatosensory perturbation has been actually reported in speech by Nasir and Ostry [3]. Future development of the model would be needed to account for their results, since Nasir and Ostry’s paradigm uses a perturbation of the jaw along the horizontal direction, making thus possible a perturbation of the somatosensory feedback without inducing changes in the auditory domain.

More generally, the present model provides a powerful framework for testing hypotheses on the relative roles of auditory and somatosensory representations and processes in perceptual and motor responses to perturbations. Indeed, any means likely to modulate one or the other input (e.g., by exploiting inter-individual variability—or by decreasing the salience of one modality relative to the other, by various techniques such as masking or inhibition of a given channel) should modify the amount of response to perturbations, and thus generate specific quantitative predictions to be compared with new experimental data (e.g., [73]).

Finally, it could be interesting to relate our computational framework with putative neuroanatomical networks suggested by neurocognitive data from the literature. As a matter of fact, a number of studies have explored the neuroanatomy of circuits in charge of monitoring responses to auditory or somatosensory perturbations in speech production (e.g., [74–81]). Even though this is out of the focus of the present study, we have already undertaken studies suggesting possible neuroanatomical correlates of the generic COSMO model [82], which is compatible with the current computational model. A future step in this direction is to adapt the generic architecture to the specific processes associated to perturbation compensation. This would be necessary for better addressing the dynamic adaptation processes mentioned previously in this section.

### Conclusions

In order to better understand the mechanisms underlying the observations reported by Lametti et al. [1], we have elaborated a simplified Bayesian model of speech production and speech perception in which phonemes are characterized both in somatosensory and auditory terms. Speech production is assumed to be guided by both sensory characterizations (hypothesis $Q_{\text{Prod}}^{F}$). Two hypotheses concerning speech perception processes were evaluated: (1) speech perception relies only on the auditory pathway (hypothesis $Q_{\text{Per}}^{A}$), or (2) speech perception relies on the fusion of both auditory and somatosensory pathways (hypothesis $Q_{\text{Per}}^{F}$). We have also considered different hypotheses on the possible consequences of motor adaptation: (1) an update of the auditory-motor internal model, (2) an update of the auditory characterization of the perturbed phoneme, and (3) an update of its somatosensory characterization. Taken separately or in combination, these three update hypotheses lead to seven possible adaptation hypotheses. Combined with the two perception hypotheses $Q_{\text{Per}}^{A}$ and $Q_{\text{Per}}^{F}$, these adaptation hypotheses lead to different possible scenarios for explaining the observations of the study of Lametti et al. [1].

In the context of our Bayesian model, we have compared the predictions of these possible scenarios with the experimental observations reported by Lametti et al. [1]. Considering results in perception and production, our simulations indicate that three combined perception-adaptation hypotheses can reproduce the characteristics of the perceptual boundary shift observed in L-14: (1) speech perception relies both on the somatosensory and auditory pathways, and motor adaptation induces only a local update of the auditory-motor internal model ($Q_{\text{Per}}^{F}⊕H_{\text{Ad}}^{M}$); (2) speech perception relies only on the auditory pathway and motor adaptation induces both a local update of the auditory-motor internal model and the combined shift and size reduction of the auditory characterization of the perturbed phoneme ($Q_{\text{Per}}^{A}⊕H_{\text{Ad}}^{M\Phi }$), (3) speech perception relies both on the somatosensory and auditory pathways and motor adaptation induces both a local update of the auditory-motor internal model and the combined shift and size reduction of the perturbed phoneme ($Q_{\text{Per}}^{F}⊕H_{\text{Ad}}^{M\Phi }$).

From that basis, these three selected hypotheses were further evaluated with respect to the predicted correlation between compensation in production and perceptual shift. Our results indicate that only the third hypothesis ($Q_{\text{Per}}^{F}⊕H_{\text{Ad}}^{M\Phi }$) is able to account for the absence of correlation reported by Lametti et al. [1].

Altogether, this computational approach strengthens and specifies the interpretation by Lametti et al. [1] of their experimental data in favor of perceptuo-motor links in speech perception. Our model provides novel insights into the mechanisms influencing speech perception and production after adaptation to perturbations of the auditory feedback. Future work should focus on the dynamics of adaptation as well as on the relation between the degree of adaptation and the amount of perceptual changes.

## Supporting information

S1 Text Detailed model definition. (PDF)

S2 Text Derivation of Bayesian inference equations. (PDF)

S3 Text Specification of parameters for the local update of the auditory-motor mapping *ρ*_*A*_. (PDF)

S4 Text Specification of parameters of the sensory characterizations of phonemes *P*(*A*_Φ_ | Φ) and *P*(*S*_Φ_ | Φ). (PDF)

S5 Text From L-14 to S-09: Variations around the theme. (PDF)
