# Supplementary material for: What drives the perceptual change resulting from speech motor adaptation? Evaluation of hypotheses in a Bayesian modeling framework
Source: PLoS Comput Biol. 2018 Jan 22;14(1):e1005942. doi: 10.1371/journal.pcbi.1005942 (PMC5794199; doi:10.1371/journal.pcbi.1005942)
Supplement: S1 Text — (PDF) [file pcbi.1005942.s001.pdf]

Supporting information S1. Detailed model definition

This section provides additional technical description of the way our model is defined. The model definition applies the Bayesian Programming methodology [1], that proceeds in three steps: first, variables are selected and defined, second, the joint probability distribution over these variables is defined, usually by decomposing it as a product of probability distributions, which are simplified thanks to conditional independence hypotheses, and third and last, each of the terms in the decomposition is associated to a parametric form and a manner to identify these parameters (whether by experimental learning or by a priori definitions). We now provide the definition of the model, following each of these steps.

Variable definitions

For convenience, we recall here the variable definitions provided in the main text. Variables M, A_M, S_M, A_Φ and S_Φ are one dimensional continuous variables, i.e., each is in IR. Variable Φ is a three-valued categorical variable, with $\Phi = \{/i/, /\varepsilon/, /a/\}$. Finally, variables C_S and C_A are binary variables, i.e. two-valued categorical variables, with $C_S = C_A = \{0, 1\}$.

Decomposition of the joint probability distribution

With these variables, the joint probability distribution that defines our model mathematically is $P(M\ S_M\ A_M\ \Phi\ S_\Phi\ A_\Phi\ C_S\ C_A)$. Choosing a variable ordering and applying the chain rule, it is equal to:

$$
\begin{aligned}
& P(M\ S_M\ A_M\ \Phi\ S_\Phi\ A_\Phi\ C_S\ C_A) \\
&= P(M)P(A_M \mid M)P(S_M \mid A_M\ M) \\
&\quad P(\Phi \mid S_M\ A_M\ M)P(A_\Phi \mid \Phi\ S_M\ A_M\ M)P(S_\Phi \mid A_\Phi\ \Phi\ S_M\ A_M\ M) \\
&\quad P(C_A \mid S_\Phi\ A_\Phi\ \Phi\ S_M\ A_M\ M)P(C_S \mid C_A\ S_\Phi\ A_\Phi\ \Phi\ S_M\ A_M\ M)\ .
\end{aligned} \tag{1}
$$

We now apply conditional independence hypotheses to simplify some of these terms.

The first two, $P(M)$ and $P(A_M \mid M)$, are left unchanged. The term $P(S_M \mid A_M\ M)$ is simplified into $P(S_M \mid M)$: this assumes that the cognitive agent's knowledge about the somatosensory consequence of some motor command m is independent of the acoustic consequence of m when m is known. In other words, the main cause of somatosensory signals S_M is assumed to be motor commands, and the cognitive agent dismisses the additional information carried out by A_M about S_M. What is lost in this approximation is the possible physical effect of acoustic waves provoked by sound production on somatosensory sensors; an effect likely to be negligible. It has to be noted that this conditional independence hypothesis between S_M and A_M given M does not entail at all independence between S_M and A_M. For instance, in the model, the cognitive agent can retrieve $P(S_M\ A_M) \propto \sum_M P(M)P(A_M \mid M)P(S_M \mid M)$ which is not equal to $P(S_M)P(A_M)$ in the general case. This means that the model contains knowledge about relations between auditory and somatosensory consequences of motor commands, but it does not store it as an explicit piece of knowledge.

The three next terms are assumed to constitute a separate piece of model, independent from knowledge about motor commands and their sensory consequences, so that variables S_M, A_M and M can be dropped. This yields $P(\Phi)$, $P(A_\Phi \mid \Phi)$ and $P(S_\Phi \mid A_\Phi\ \Phi)$. Furthermore, using a similar conditional independence hypothesis as above, we assume that phonemes are characterized independently into

acoustic and somatosensory spaces. In other words, the somatosensory characterization S_Φ of some phoneme ϕ is supposed to be independent of the acoustic characterization A_Φ of this phoneme, when ϕ is known. Therefore, $P(S_\Phi \mid A_\Phi\ \Phi)$ is simplified into $P(S_\Phi \mid \Phi)$.

Finally, the last two terms concerns coherence variables, which we, as modelers, connect explicitly to chosen variables: first, variable C_A serves as a connector between auditory representations A_M and A_Φ, second, variable C_S serves as a connector between somatosensory representations S_M and S_Φ. This yields terms $P(C_A \mid A_M\ A_\Phi)$ and $P(C_S \mid S_M\ S_\Phi)$.

Replacing each term of Eq (1) by its simplified form, we obtain the decomposition of the joint distribution shown in the main text (Eq. (1)), which we repeat here:

$$\begin{aligned} & P(M\ S_M\ A_M\ \Phi\ S_\Phi\ A_\Phi\ C_S\ C_A) \\ = \quad & P(M)P(A_M \mid M)P(S_M \mid M) \\ & P(\Phi)P(A_\Phi \mid \Phi)P(S_\Phi \mid \Phi) \\ & P(C_A \mid A_M\ A_\Phi)P(C_S \mid S_M\ S_\Phi)\ . \end{aligned} \tag{2}$$

Parametric forms

Parametric forms for all terms in Eq (2) are provided in the main text. We still describe here, in a bit more detail, the properties of coherence variables (demonstrations are available elsewhere [2, 1]. Recall that coherence variables are binary variables associated with Dirac distributions that enforce matching constraints. Consider C_A: we have defined

$$P\left([C_A = 1] \mid [A_M = a_m]\ [A_\Phi = a_\phi]\right) \quad := \quad \begin{cases} 1 & \text{if } a_m = a_\phi; \\ 0 & \text{otherwise.} \end{cases} \tag{3}$$

Given this definition, coherence variables can be used, during inference, as “switches”, allowing the modeler to control the propagation of information throughout the model. There are three cases to consider.

First, when left unspecified in the computed question, the switch is open, and portions of the model on each side of the coherence variable do not exchange information. For instance, in the model, the portion of the model about phoneme characterizations can be “separated” from the portion about the sensory consequences of motor commands: $P(M \mid A_M\ S_M)$ can be completely computed by involving terms $P(M)$, $P(A_M \mid M)$ and $P(S_M \mid M)$, as other terms of the decomposition are “beyond” the coherence variables, which are not specified in $P(M \mid A_M\ S_M)$. Computing the motor cause of some sensed sensory event A_M, S_M would only involve knowledge about the way motor commands provoke sensory effects; whatever the phonological plausibility of this sensory event.

Second, when set to 1 in the computed question, the switch is closed, and variables on each side of the coherence variable are forced to have equal values, so that information about one variable propagates and constrains the other. For instance, in the model, computing $P(M \mid A_M\ S_M\ [C_A = 1]\ [C_S = 1])$ would be influenced by phonological knowledge, as the “switches” are here closed so that A_M is constrained by A_Φ and S_M is constrained by S_Φ. Here, computing the motor cause of some sensed sensory event, A_M and S_M, would also involve the phonological plausibility of this sensory event.

Third and finally, when set to 0, variables are forced to be different, which is less useful in practice – but see [1, p 139].

References

1. Bessière P, Mazer E, Ahuactzin JM, Mekhnacha K. Bayesian Programming. Boca Raton, Florida: CRC Press; 2013.

2. Gilet E, Diard J, Bessière P. Bayesian Action–Perception Computational Model: Interaction of Production and Recognition of Cursive Letters. PLOS ONE. 2011;6(6):e20387.
