# Supplementary material for: What drives the perceptual change resulting from speech motor adaptation? Evaluation of hypotheses in a Bayesian modeling framework
Source: PLoS Comput Biol. 2018 Jan 22;14(1):e1005942. doi: 10.1371/journal.pcbi.1005942 (PMC5794199; doi:10.1371/journal.pcbi.1005942)
Supplement: S2 Text — (PDF) [file pcbi.1005942.s002.pdf]

Supporting information S2.
Derivation of Bayesian inference equations

Here we detail to derivation of Eqs (8) to (13) of the main text. All computations rest on the same principle, i.e. the application of Bayesian inference. Bayes rule dictates how to compute conditional probabilities when the joint probability distribution of variables is known. For instance, in the case of two probabilistic variables A and B, the conditional probability $P(A \mid B)$ is computed from the joint probability $P(A\ B)$ as (provided that $P(B) \neq 0$):

$$P(A \mid B) = \frac{P(A\ B)}{P(B)} = \frac{P(A\ B)}{\sum_{A} P(A\ B)}.$$

This is generalized to $(n+1)$ variables, $\{A_1, A_2, \cdots, A_n, B\}$ as:

$$P(A_1 \mid B) = \frac{P(A_1\ B)}{P(B)} = \frac{\sum_{/\{A_1,B\}} P(A_1\ \cdots A_n\ B)}{\sum_{/\{B\}} P(A_1\ \cdots A_n\ B)}, \tag{1}$$

where $\sum_{/X}$ denotes summing over all variables but those included in the set X.

We will also consider conditional probabilities of more than two variables, which are obtained in a similar way:

$$P(A_1 \mid A_2\ B) = \frac{P(A_1\ A_2\ B)}{P(A_2\ B)} = \frac{\sum_{/\{A_1,A_2,B\}} P(A_1 \cdots A_n\ B)}{\sum_{/\{A_2,B\}} P(A_1 \cdots A_n\ B)}, \tag{2}$$

$$P(A_1 \mid A_2\ A_3\ B) = \frac{P(A_1\ A_2\ A_3\ B)}{P(A_2\ A_3\ B)} = \frac{\sum_{/\{A_1,A_2,A_3,B\}} P(A_1 \cdots A_n\ B)}{\sum_{/\{A_2,A_3,B\}} P(A_1 \cdots A_n\ B)}. \tag{3}$$

Independence of M and Φ.

The first result to demonstrate concerns the conditional independence between M and Φ. We have to show that the model predicts $P(M \mid \Phi) = P(M)$. This result is obtained by computing $P(M \mid \Phi)$ from the joint probability distribution $P(M\ S_M\ A_M\ \Phi\ S_\Phi\ A_\Phi\ C_S\ C_A)$, using Bayes rule in Eq (1) :

$$P(M \mid \Phi) = \frac{P(M\ \Phi)}{P(\Phi)} = \frac{\sum_{/\{M,\Phi\}} P(M\ S_M\ A_M\ \Phi\ S_\Phi\ A_\Phi\ C_S\ C_A)}{\sum_{/\{\Phi\}} P(M\ S_M\ A_M\ \Phi\ S_\Phi\ A_\Phi\ C_S\ C_A)}. \tag{4}$$

In order to avoid confusions, we draw attention to the notation that is employed here. The domain of the joint probability distribution is composed of discrete and continuous variables. Usually, one writes P for probability distributions over discrete variables and p for probability densities over continuous variables. For simplicity, we chose not to make this distinction here. Similarly, all summations and integrals are denoted by the sign $\sum$, even when rigorously it is the $\int$ sign that should be used for continous variables.

The summations in Eq (4) can be performed by replacing the joint probability distribution by its decomposition:

$$\begin{aligned} P(M\ S_M\ A_M\ \Phi\ S_\Phi\ A_\Phi\ C_S\ C_A) &= P(M)P(A_M \mid M)P(S_M \mid M) \\ &\quad P(\Phi)P(A_\Phi \mid \Phi)P(S_\Phi \mid \Phi) \\ &\quad P(C_A \mid A_M\ A_\Phi)P(C_S \mid S_M\ S_\Phi). \end{aligned} \tag{5}$$

Since probability distributions are normalized, reorganizing the sums in the numerator leads to unit factors, and only the product $P(M)P(\Phi)$ remains. The same holds for the denominator, where in addition the sum over M also reduces the factor $P(M)$ to 1, and hence only $P(\Phi)$ remains. Altogether, Eq (4) becomes:

$$P(M \mid \Phi) = \frac{P(M)P(\Phi)}{P(\Phi)} = P(M), \tag{6}$$

proving that M is independent of Φ.

Similar steps enable to prove that variables Φ and A_M are also independent.

Production questions.

Three production questions were defined in the text. They corresponded to the planning of motor commands M for the production of phoneme Φ, considering that either C_A, C_S, or both, are set to 1.

The first and second questions, $P(M \mid \Phi\ [C_A = 1])$ and $P(M \mid \Phi\ [C_S = 1])$, are computed in the same way using Eq (2). We only detail here the computations in the case of $P(M \mid \Phi\ [C_A = 1])$. From Eq (2) we have:

$$P(M \mid \Phi\ [C_A = 1]) \propto \sum_{/\{M,\Phi,C_A\}} P(M\ S_M\ A_M\ \Phi\ S_\Phi\ A_\Phi\ C_S\ [C_A = 1]), \tag{7}$$

in which the denominator, independent of M, has been included in the proportionality symbol $\propto$. The summation in Eq (7) is performed using the decomposition of the joint probability distribution in Eq (1) in the text. Again, most terms sum to 1, such that Eq (7) results in:

$$P(M \mid \Phi\ [C_A = 1]) \propto \sum_{A_M, A_\Phi} P(A_M \mid M)P(A_\Phi \mid \Phi)P([C_A = 1] \mid A_\Phi\ A_M), \tag{8}$$

where $P(M)$ and $P(\Phi)$ were also included in the proportionality symbol since they are assumed to be uniform. $P(A_M \mid M)$ in Eq (8) is a Dirac delta function given by:

$$P([A_M = a] \mid [M = m]) := \delta\left(a - \rho_A(m)\right), \tag{9}$$

such that, in the sum over values taken by A_M in Eq (8), only the term involving value $a = \rho_A(m)$ does not vanish. Eq (8) becomes:

$$P([M = m] \mid \Phi\ [C_A = 1]) \propto \sum_{A_\Phi} P(A_\Phi \mid \Phi)P([C_A = 1] \mid A_\Phi\ [A_M = \rho_A(m)]). \tag{10}$$

The second factor in Eq (10) corresponds to the sensory-matching constraint defined as:

$$P\left([C_A = 1] \mid [A_M = a_m]\ [A_\Phi = a_\phi]\right) := \begin{cases} 1 & \text{if } a_m = a_\phi; \\ 0 & \text{otherwise.} \end{cases} \tag{11}$$

Hence, in the sum over values taken by A_Φ in Eq (10), only the term involving value $\rho_A(m)$ does not vanish. Hence Eq (10) becomes:

$$\boxed{P([M = m] \mid \Phi\ [C_A = 1]) \propto P([A_\Phi = \rho_A(m)] \mid \Phi).} \tag{12}$$

The third question, $P(M \mid \Phi\ [C_A = 1][C_S = 1])$, is computed from Eq (3):

$$P(M \mid \Phi\ [C_A=1]\ [C_S=1]) \propto \sum_{/\{M,\Phi,C_A,C_S\}} P(M\ S_M\ A_M\ \Phi\ S_\Phi\ A_\Phi\ [C_S=1]\ [C_A=1]), \tag{13}$$

in which again, the denominator, being independent of M, has been included in the proportionality symbol $\propto$. As before, the summation in Eq (13) is performed using the decomposition of the joint probability distribution in Eq (5), resulting in:

$$\begin{aligned} &P(M \mid \Phi\ [C_A=1]\ [C_S=1]) \\ &\propto \sum_{\{A_M,A_\Phi\}} P(A_M \mid M) P(A_\Phi \mid \Phi) P([C_A=1] \mid A_\Phi\ A_M) \\ &\quad \sum_{\{S_M,S\Phi\}} P(S_M \mid M) P(S_\Phi \mid \Phi) P([C_S=1] \mid S_\Phi\ S_M) \end{aligned} \tag{14}$$

where again $P(M)$ and $P(\Phi)$ were included in the proportionality symbol since they are assumed to be uniform.

As before, in the sum over values taken by A_M and S_M in Eq (14), only the terms involving values $a = \rho_A(m)$ and $s = \rho_S(m)$ do not vanish. Hence Eq (14) becomes:

$$\begin{aligned} &P([M=m] \mid \Phi\ [C_A=1]\ [C_S=1]) \\ &\propto \sum_{A_\Phi} P(A_\Phi \mid \Phi) P([C_A=1] \mid A_\Phi\ [A_M=\rho_A(m)]) \\ &\quad \sum_{S_\Phi} P(S_\Phi \mid \Phi) P([C_S=1] \mid S_\Phi\ [S_M=\rho_S(m)]). \end{aligned} \tag{15}$$

Then again, with the definition of the two sensory matching constraints, in the sum over values taken by A_Φ and S_Φ in Eq (15), only terms involving values $\rho_A(m)$ $\rho_S(m)$ do not vanish. Hence Eq (15) becomes:

$$\boxed{P([M=m] \mid \Phi\ [C_A=1]\ [C_S=1]) \propto P([A_\Phi=\rho_A(m)] \mid \Phi) P([S_\Phi=\rho_S(m)] \mid \Phi).} \tag{16}$$

Perception questions.

Three perception questions were defined in the text. They corresponded to the inference of phoneme identity Φ given the auditory input A_M, considering that either C_A, C_S, or both, are set to 1.

The first question, $P(\Phi \mid A_M\ [C_A=1])$ is computed from Eq (2):

$$P(\Phi \mid A_M\ [C_A=1]) = \frac{\sum_{/\{A_M,\Phi,C_A\}} P(M\ S_M\ A_M\ \Phi\ S_\Phi\ A_\Phi\ C_S\ [C_A=1])}{\sum_{/\{A_M,C_A\}} P(M\ S_M\ A_M\ \Phi\ S_\Phi\ A_\Phi\ C_S\ [C_A=1])}. \tag{17}$$

The decomposition of the joint probability distribution (Eq (5)) is used to perform the sums in Eq (17). Sums over C_S, S_M and S_Φ reduce to factors of 1, such that:

$$\begin{aligned} &P(\Phi \mid [A_M=a]\ [C_A=1]) \\ &= \frac{\sum_{M,A_\Phi} P([A_M=a] \mid M) P(A_\Phi \mid \Phi) P([C_A=1] \mid A_\Phi\ [A_M=a])}{\sum_{\Phi,M,A_\Phi} P([A_M=a] \mid M) P(A_\Phi \mid \Phi) P([C_A=1] \mid A_\Phi\ [A_M=a])}, \end{aligned} \tag{18}$$

were terms $P(M)$ and $P(\Phi)$ are taken outside of the summations, since they are assumed to be constant, and further simplified since they appear both in the numerator and the denominator.

Performing the sums over A_Φ and reorganizing terms leads to the following steps:

$$
\begin{aligned}
P(\Phi \mid [A_M = a]\ [C_A = 1]) &= \frac{\sum\limits_{M} P([A_M = a] \mid M) P([A_\Phi = a] \mid \Phi)}{\sum\limits_{\Phi, M} P([A_M = a] \mid M) P([A_\Phi = a] \mid \Phi)}, \\
&= \frac{P([A_\Phi = a] | \Phi) \sum\limits_{M} P([A_M = a] \mid M)}{\sum\limits_{\Phi} P([A_\Phi = a] | \Phi) \sum\limits_{M} P([A_M = a] \mid M)}. \qquad (19)
\end{aligned}
$$

Finally, simplifying terms on the numerator and denominator of Eq (19) leads to:

$$
\boxed{P(\Phi \mid [A_M = a]\ [C_A = 1]) = \frac{P([A_\Phi = a] \mid \Phi)}{\sum\limits_{\Phi} P([A_\Phi = a] \mid \Phi)}.} \qquad (20)
$$

The second perception question, $P(\Phi \mid A_M\ [C_S = 1])$, is also computed from Eq (2).

$$
P(\Phi \mid A_M\ [C_S = 1]) = \frac{\sum\limits_{/\{A_M, \Phi, C_S\}} P(M\ S_M\ A_M\ \Phi\ S_\Phi\ A_\Phi\ C_A\ [C_S = 1])}{\sum\limits_{/\{A_M, C_S\}} P(M\ S_M\ A_M\ \Phi\ S_\Phi\ A_\Phi\ C_A\ [C_S = 1])}. \qquad (21)
$$

Again, using the decomposition of the joint probability distribution enables to perform the sums in Eq (21). The sums over A_Φ and C_A reduce to 1 such that:

$$
\begin{aligned}
&P(\Phi \mid [A_M = a]\ [C_S = 1]) \\
&= \frac{\sum\limits_{M, S_\Phi, S_M} P([A_M = a] \mid M) P(S_M \mid M) P(S_\Phi \mid \Phi) P([C_S = 1] \mid S_\Phi\ S_M)}{\sum\limits_{\Phi, M, S_\Phi, S_M} P([A_M = a] \mid M) P(S_M \mid M) P(S_\Phi \mid \Phi) P([C_S = 1] \mid S_\Phi\ S_M)}, \qquad (22)
\end{aligned}
$$

were terms $P(M)$ and $P(\Phi)$ were again taken outside of the sums, since they are assumed to be constant, and further simplified.

In the sums over M in Eq (22), the factor $P([A_M = a] \mid M)$ is zero unless M takes a value for which its image through the auditory-motor mapping ρ_A is a. In other words, the sum is reduced to the set of inverse images of a, $m_a \in \{\rho_A^{-1}(a)\}$, and Eq (22) becomes :

$$
\begin{aligned}
&P(\Phi \mid [A_M = a]\ [C_S = 1]) \\
&= \frac{\sum\limits_{m_a \in \{\rho_A^{-1}(a)\}} \sum\limits_{S_\Phi, S_M} P(S_M \mid [M = m_a]) P(S_\Phi \mid \Phi) P([C_S = 1] \mid S_\Phi\ S_M)}{\sum\limits_{m_a \in \{\rho_A^{-1}(a)\}} \sum\limits_{\Phi, S_\Phi, S_M} P(S_M \mid [M = m_a]) P(S_\Phi \mid \Phi) P([C_S = 1] \mid S_\Phi\ S_M)}. \qquad (23)
\end{aligned}
$$

Next, in the sums over S_M, because of the Dirac delta function $P(S_M | [M = m_a])$, only the term $S_M = \rho_S(m_a)$ remains, hence Eq (23) becomes:

$$
\begin{aligned}
&P(\Phi \mid [A_M = a]\ [C_S = 1]) \\
&= \frac{\sum\limits_{m_a \in \{\rho_A^{-1}(a)\}} \sum\limits_{S_\Phi} P(S_\Phi \mid \Phi) P([C_S = 1] \mid S_\Phi\ [S_M = \rho_S(m_a)])}{\sum\limits_{m_a \in \{\rho_A^{-1}(a)\}} \sum\limits_{\Phi, S_\Phi} P(S_\Phi \mid \Phi) P([C_S = 1] \mid S_\Phi\ [S_M = \rho_S(m_a)])}. \qquad (24)
\end{aligned}
$$

Finally, performing the sums over S_Φ, because of the sensory matching constraint $P([C_S = 1] \mid S_\Phi\ [S_M = \rho_S(m_a)])$, we obtain:

$$
P(\Phi \mid [A_M = a]\ [C_S = 1]) = \frac{\sum\limits_{m_a \in \{\rho_A^{-1}(a)\}} P([S_\Phi = \rho_S(m_a)] \mid \Phi)}{\sum\limits_{m_a \in \{\rho_A^{-1}(a)\}} \sum\limits_{\Phi} P([S_\Phi = \rho_S(m_a)] \mid \Phi)}. \qquad (25)
$$

For an injective auditory-motor mapping ρ_A, as it is the case in normal conditions or in the global update hypothesis (linear mapping), auditory values a have a unique inverse image which is directly found as $a_m = \rho_A^{-1}(a)$. In this case the sum is reduced to a unique term and Eq (25) becomes:

$$\boxed{P(\Phi \mid [A_M = a]\ [C_S = 1]) = \frac{P([S_\Phi = \rho_S \circ \rho_A^{-1}(a)] \mid \Phi)}{\sum\limits_{\Phi} P([S_\Phi = \rho_S \circ \rho_A^{-1}(a)] \mid \Phi)}.} \tag{26}$$

In the local update hypothesis, the auditory-motor mapping is no longer injective. In Supplementary information S3 we indicate how the different inverse images are found.

The third perception question, $P(\Phi \mid A_M\ [C_A = 1][C_S = 1])$ is computed from Eq (3). The steps are similar to the previous cases and lead to:

$$\begin{aligned} &P(\Phi \mid [A_M = a]\ [C_S = 1]) \\ &\quad = \frac{P([A_\Phi = a] \mid \Phi) \sum\limits_{m_a \in \{\rho_A^{-1}(a)\}} P([S_\Phi = \rho_S(m_a)] \mid \Phi)}{\sum\limits_{m_a \in \{\rho_A^{-1}(a)\}} \sum\limits_{\Phi} P([A_\Phi = a] \mid \Phi) P([S_\Phi = \rho_S(m_a)] \mid \Phi)}, \end{aligned} \tag{27}$$

which again, for injective auditory-motor mappings ρ_A, as in normal conditions or in the global update hypothesis, results in:

$$\boxed{P(\Phi \mid [A_M = a]\ [C_S = 1]) = \frac{P([A_\Phi = a] \mid \Phi) P([S_\Phi = \rho_S \circ \rho_A^{-1}(a)] \mid \Phi)}{\sum\limits_{\Phi} P([A_\Phi = a] \mid \Phi) P([S_\Phi = \rho_S \circ \rho_A^{-1}(a)] \mid \Phi)}.} \tag{28}$$
