# Supplementary material for: What drives the perceptual change resulting from speech motor adaptation? Evaluation of hypotheses in a Bayesian modeling framework
Source: PLoS Comput Biol. 2018 Jan 22;14(1):e1005942. doi: 10.1371/journal.pcbi.1005942 (PMC5794199; doi:10.1371/journal.pcbi.1005942)
Supplement: S3 Text — (PDF) [file pcbi.1005942.s003.pdf]

Supporting information S3. Specification of parameters for the local update of the auditory-motor mapping ρ_A

In the local update hypothesis, the linear relation assumed for the auditory-motor mapping in normal condition must be abandoned. For computational simplicity, we assume that the new auditory-motor internal model is linear in a piece-wise manner, as illustrated in Fig 1 and given by:

$$\rho_A^{(u)}(m) := \begin{cases} \rho_1(m) = m + \delta_A & \text{if } m \in [\eta - \omega \ ; \ \eta + \omega], \\ \rho_2(m) = \frac{\chi + \delta_A}{\chi} m + \delta_A \left(1 - \frac{\eta - \omega}{\chi}\right) & \text{if } m \in [\eta - \omega - \chi \ ; \ \eta - \omega), \\ \rho_3(m) = \frac{\chi - \delta_A}{\chi} m + \delta_A \left(1 + \frac{\eta + \omega}{\chi}\right) & \text{if } m \in \ (\eta + \omega \ ; \ \eta + \omega + \chi], \\ \rho_4(m) = m & \text{otherwise.} \end{cases} \tag{1}$$

Parameter δ_A corresponds to the magnitude of the update (equal to the perturbation in the case where full compensation is assumed). Parameters η and ω correspond respectively to the center and width of the updated region in the motor command domain. Parameter χ specifies the width in the motor command domain of the intermediate intervals joining the updated and non-updated intervals of the mapping. Note that the general update is recovered when $\chi \to +\infty$.

Eq (1) and Fig 1 allow to identify the inverse images of an auditory output a. Depending on the sign of $\delta_A - \chi$ and on the location of a there is one, two or three inverse images (we leave aside the case where $\delta_A = \chi$ for which there is an infinite number of inverse images corresponding to the segment $[\eta + \omega; \eta + \omega + \chi]$):

If $\delta_A > \chi$ and $a \in [(\eta + \omega + \chi) \ ; \ (\eta + \omega + \delta_A)]$, there are three inverse images, corresponding to:

$$\begin{cases} m_1 &= \begin{cases} \rho_2^{-1}(a) = \frac{\chi}{\chi + \delta_A}\left(a - \delta_A \left(1 - \frac{\eta - \omega}{\chi}\right)\right) & \text{if } a \in [(\eta + \omega + \chi) \ ; \ (\eta - \omega + \delta_A)], \\ \rho_1^{-1}(a) = a - \delta_A & \text{otherwise,} \end{cases} \\ m_2 &= \rho_3^{-1}(a) = \frac{\chi}{\chi - \delta_A}\left(a - \delta_A \left(1 + \frac{\eta + \omega}{\chi}\right)\right), \\ m_3 &= \rho_4^{-1}(a) = a. \end{cases} \tag{2}$$

If $\delta_A > \chi$ and $a = \eta + \omega + \chi$, there are two inverse images, corresponding to:

$$\begin{cases} m_1 &= \begin{cases} \rho_2^{-1}(a) = \frac{\chi}{\chi + \delta_A}\left(a - \delta_A \left(1 - \frac{\eta - \omega}{\chi}\right)\right) & \text{if } (\eta + \omega + \chi) > (\eta - \omega + \delta_A), \\ \rho_1^{-1}(a) = a - \delta_A & \text{otherwise,} \end{cases} \\ m_2 &= \rho_4^{-1}(a) = \eta + \omega + \chi. \end{cases} \tag{3}$$

If $\delta_A < \chi$ or $a \notin [(\eta + \omega + \chi) \ ; \ (\eta + \omega + \delta_A)]$, there is a single inverse image corresponding to:

$$m = \begin{cases} \rho_1^{-1}(a) = a - \delta_A & \text{if } a \in \ [(\eta - \omega + \delta_A) \ ; \ (\eta + \omega + \chi)], \\ \rho_2^{-1}(a) = \frac{\chi}{\chi + \delta_A}\left(a - \delta_A \left(1 - \frac{\eta - \omega}{\chi}\right)\right) & \text{if } a \in \ [(\eta - \omega - \chi) \ ; \\ & \quad \min\left((\eta + \omega + \chi), (\eta - \omega + \delta_A)\right)], \\ \rho_4^{-1}(a) = a & \text{otherwise.} \end{cases} \tag{4}$$

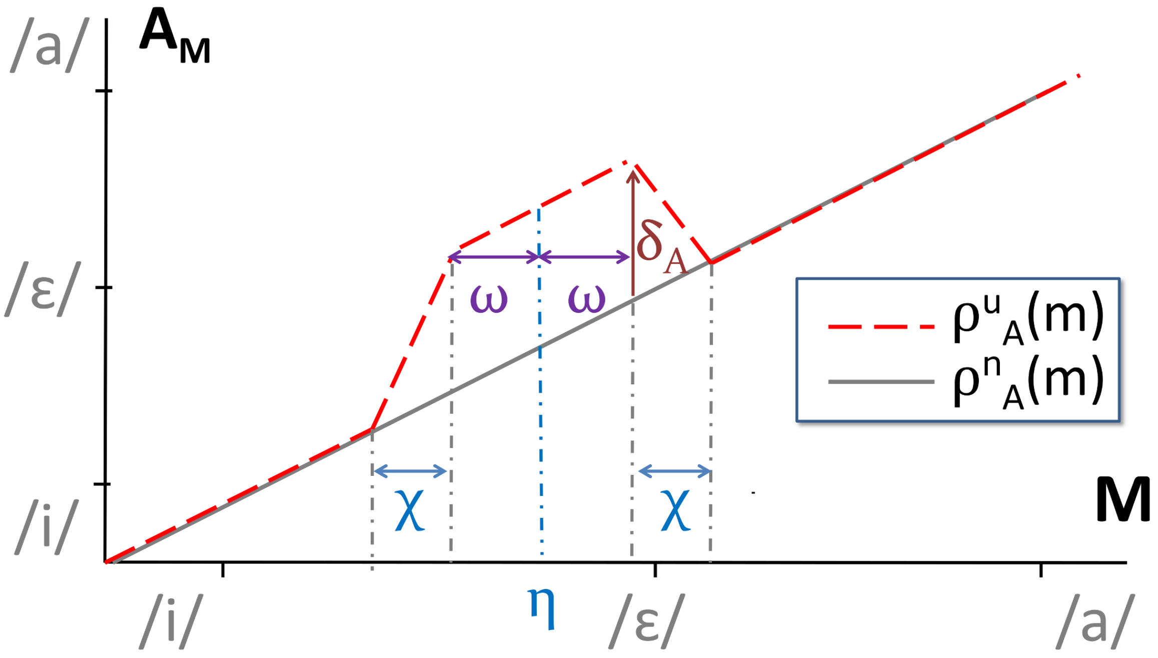

Figure 1: Auditory-motor mapping ρ_A before and after a local update. The plain gray straight line corresponds to $\rho_A^{(n)}$, the linear auditory-motor mapping in normal condition. The red dashed line corresponds to $\rho_A^{(u)}$, the locally updated auditory-motor mapping. Parameters η and ω correspond to the center and width, in the motor command domain, of the updated portion of the internal model. Parameter χ specifies the width, in the motor command domain, of the intermediate interval joining the updated and non-updated intervals of the mapping.
