# Supplementary material for: What drives the perceptual change resulting from speech motor adaptation? Evaluation of hypotheses in a Bayesian modeling framework
Source: PLoS Comput Biol. 2018 Jan 22;14(1):e1005942. doi: 10.1371/journal.pcbi.1005942 (PMC5794199; doi:10.1371/journal.pcbi.1005942)
Supplement: S4 Text — (PDF) [file pcbi.1005942.s004.pdf]

Supporting information S4. Specification of parameters of the sensory characterizations of phonemes $P(A_\Phi \mid \Phi)$ and $P(S_\Phi \mid \Phi)$

S4.1. Normal condition.

The sensory characterization of phonemes are characterized by parameters $(\mu_A^\Phi, \sigma_A^\phi)$ and $(\mu_S^\Phi, \sigma_S^\phi)$. Phonemes are assumed to be equally spaced in both sensory spaces. In addition, units of auditory and somatosensory spaces are defined such that the distance between two neighboring phonemes is 1. Hence, in normal conditions the center of each sensory characterization is defined by:

$$\mu_A^{i\,(n)} = \mu_S^{i\,(n)} = 1, \tag{1}$$

$$\mu_A^{\varepsilon\,(n)} = \mu_S^{\varepsilon\,(n)} = 2, \tag{2}$$

$$\mu_A^{a\,(n)} = \mu_S^{a\,(n)} = 3. \tag{3}$$

In addition, in normal conditions all sensory characterizations are assumed to have same standard deviation, equal to $\frac{1}{8}$ of the distance between neighboring phonemes. In other words:

$$\sigma_S^{\phi\,(n)} = \sigma_A^{\phi\,(n)} = \frac{1}{8}, \qquad \phi \in \{\text{ i, ε, a }\}. \tag{4}$$

S4.2. Adapted condition: relation between parameters μ_A^ε and σ_A^ε.

In the text we suggested that choosing a correct combination of parameters μ_A and σ_A could reproduce the observation reported in L-14 by canceling out the boundary shift induced by each of the parameter updates on one side of the auditory space. Here we derive the relation that was used in simulations involving this combined update of μ_A and σ_A.

In the case of a perturbation of vowel /ε/ toward vowel /a/, the boundary shift was reported to be significant in the portion of auditory space between vowel /i/ and /ε/ and not significant in the portion of auditory space between vowels /ε/ and /a/. In order to reproduce this observation, we need to find a relation between parameters μ_A^ε and σ_A^ε that leaves unchanged the location of the boundary between vowels /ε/ and /a/. The location of this boundary correspond to the auditory value a_b for which the two corresponding auditory characterizations are equal, in other words, a_b satisfies:

$$P([A_\Phi = a_b] \mid [\Phi = /\varepsilon/]) \quad = \quad P([A_\Phi = a_b] \mid [\Phi = /a/]). \tag{5}$$

Since auditory characterizations are assumed to be normal distributions, Eq (5) becomes:

$$\mathcal{N}(a_b; \mu_A^\varepsilon, \sigma_A^\varepsilon) = \mathcal{N}(a_b; \mu_A^a, \sigma_A^a), \tag{6}$$

$$\Rightarrow \quad \frac{1}{\sqrt{2\pi}\sigma_A^\epsilon} e^{-\frac{(a_b - \mu_A^\epsilon)^2}{2{\sigma_A^\epsilon}^2}} = \frac{1}{\sqrt{2\pi}\sigma_A^a} e^{-\frac{(a_b - \mu_A^a)^2}{2{\sigma_A^a}^2}}. \tag{7}$$

Taking the logarithm of Eq (7) allows to find the desired relation:

$$\log \sigma_A^\epsilon + \frac{(a_b - \mu_A^\epsilon)^2}{2{\sigma_A^\epsilon}^2} \quad = \quad \log \sigma_A^a + \frac{(a_b - \mu_A^a)^2}{2{\sigma_A^a}^2}, \tag{8}$$

$$\Rightarrow \mu_A^\epsilon \quad = \quad a_b \pm \sigma_A^\epsilon \sqrt{2 \log \frac{\sigma_A^a}{\sigma_A^\epsilon} + \frac{(a_b - \mu_A^a)^2}{{\sigma_A^a}^2}}. \tag{9}$$

Since the boundary we are interested in is located between μ_A^ϵ and μ_A^a, among the two solutions given by Eq (9), only the one for which $\mu_A^\epsilon < a_b$ is correct. Therefore:

$$\boxed{\mu_A^\epsilon = a_b - \sigma_A^\epsilon \sqrt{2 \log \frac{\sigma_A^a}{\sigma_A^\epsilon} + \frac{(a_b - \mu_A^a)^2}{{\sigma_A^a}^2}}.} \tag{10}$$

The value of a_b is obtained from the position of the auditory characterizations in normal conditions. Since auditory characterizations have the same variance in the normal condition, a_b is simply located at half the distance between the centers of the /ɛ-a/ region in normal condition. In other words:

$$a_b = \frac{{\mu_A^{\varepsilon}}^{(n)} + {\mu_A^a}^{(n)}}{2} = \frac{2+3}{2} = \frac{5}{2}. \tag{11}$$

Of course, the relation of Eq (10) does not need to be interpreted as a precise, necessary condition for our results to hold. We do not propose, either, that it is a property of the cognitive system of phoneme representation and learning. In other words, we do not claim that Eq (10) has to be satisfied or is a property of how phonemes representations are updated. Instead, we just computed the relation between the updated parameters μ_A^ε and σ_A^ε such that the boundary between phonemes /a/ and /ɛ/ do not move at all. It is quite likely that repeated exposure to a shifted /ɛ/ will both decrease variance (as new samples have no variability) and displace the mean (towards the new location). These two mechanisms could exactly follow Eq (10), in which case the boundary would exactly not move. But, it is much more likely that they do not perfectly cancel out; in the experimental data, the boundary position probably moved, maybe under the measurement precision, and, in all likelihood, it moved less than in other experimental conditions.
