# Supplementary material for: What drives the perceptual change resulting from speech motor adaptation? Evaluation of hypotheses in a Bayesian modeling framework
Source: PLoS Comput Biol. 2018 Jan 22;14(1):e1005942. doi: 10.1371/journal.pcbi.1005942 (PMC5794199; doi:10.1371/journal.pcbi.1005942)
Supplement: S5 Text — (PDF) [file pcbi.1005942.s005.pdf]

Supporting information S5.
From L-14 to S-09: variations around the theme.

An apparent contradiction remains between the experimental data of S-09 and L-14. In L-14, the perceptual boundary shift resulting from motor learning occurs in the auditory region related to the adapted utterances of the subjects. In S-09 it occurs in the auditory region corresponding to what subjects hear during adaptation. This discrepancy was pointed out in L-14 but it was suggested that a possible explanation could lie in differences between sibilants, used in S-09, and vowels, used in L-14.

We have shown that our model is able to account for observations in L-14. Slight differences in the way the auditory-motor internal model and/or the auditory characterization of vowel /ε/ are updated could enable the prediction of observations in S-09. This is illustrated by the results presented in Fig 1, in the context of our three retained hypotheses.

On the one hand, if motor learning induces only a local update of the auditory-motor internal model in the context of a speech perception process involving the fusion of sensory pathways (Hypothesis $Q^F_{\text{Per}} \oplus H^M_{\text{Ad}}$), a slight displacement of the motor region affected by this local update in the direction of the auditory perturbation (Fig 1, top panel) would predict observations in S-09. This could be due to the fact that the extent of the articulatory changes due to compensation for the auditory perturbation is less important than in L-14. Such a difference in values of the model parameters predicting observations in L-14 is consistent with the fact that in S-09 the articulation of the fricative /s/ is at the front boundary of the articulatory space, which intrinsically limits the magnitude of the articulatory changes in the front direction, while the vowel /ε/ used in L-14 is articulated in the center of the articulatory space.

On the other hand, if motor learning induces both motor and auditory updates in the context of a pure auditory speech perception process (Hypothesis $Q^A_{\text{Per}} \oplus H^{M\Phi}_{\text{Ad}}$), shifting the auditory characterization of the perturbed phoneme combined with an insufficient narrowing (so that the opposite effects on the boundary shift would not cancel each other) would predict observations reported in S-09 (Fig 1, middle horizontal panel). Finally, combining both differences in the updates of the auditory-motor internal model and the auditory characterization of the perturbed phoneme would also predict observations in S-09 under hypothesis $Q^F_{\text{Per}} \oplus H^{M\Phi}_{\text{Ad}}$ (Fig 1, lower panel). Hence, in the context of our model, both observations in S-09 and L-14 could be explained by the same influences of motor learning on speech perception. Their differences would not be contradictory, they would only show that the two tasks induced differences in the amplitudes of the updates associated with motor learning.

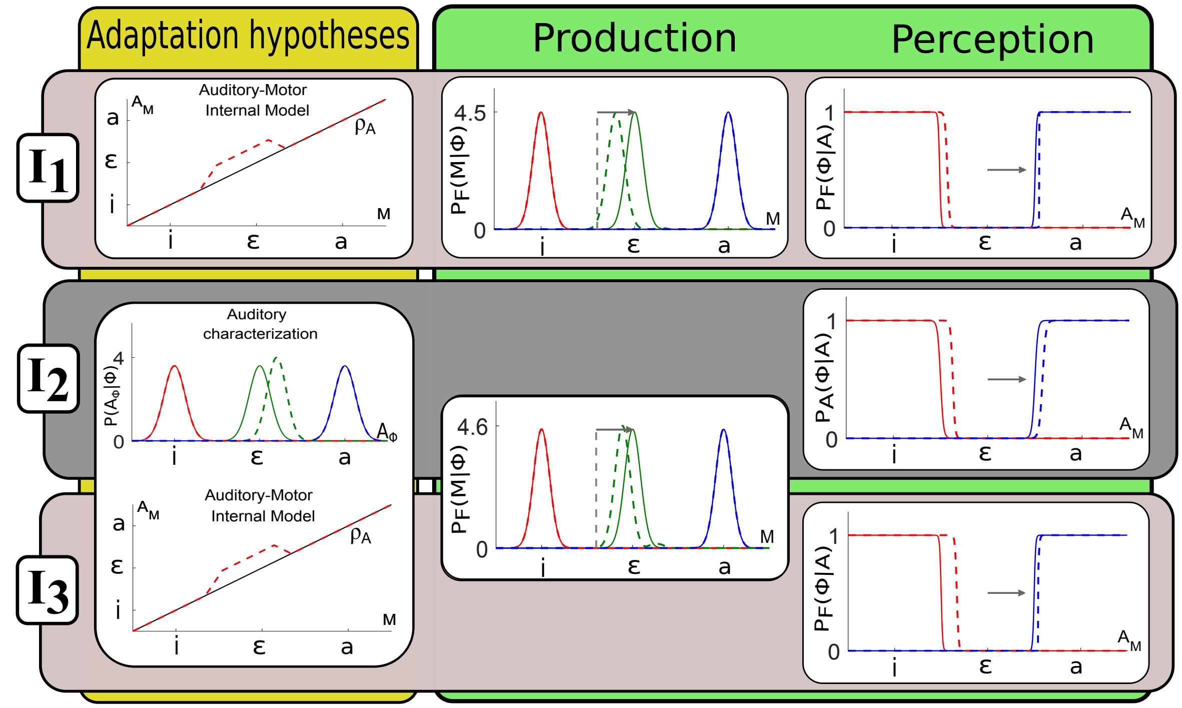

Figure 1: Proposed values of model parameters selected to account for perceptual boundary shifts on both sides of vowel /ε/ along the /i-a/ continuum. Each of the three mechanisms proposed above in the paper is able to account for such boundary shifts. For $Q^F_{\text{Per}} \oplus H^M_{\text{Ad}}$, if the locality of update of the internal model extends more to the /ε-a/ continuum compared to our previous simulations, then the perceptual shift also extents to this portion of the space. In $Q^A_{\text{Per}} \oplus H^{M\Phi}_{\text{Ad}}$ and $Q^F_{\text{Per}} \oplus H^{M\Phi}_{\text{Ad}}$, a perceptual boundary shift is obtained in both sides of vowel /ε/ along the auditory continuum with same shift but smaller narrowing than in previous simulations.
